# Matrix metalloproteinase 9: An emerging biomarker for classification of adherent vestibular schwannoma

**DOI:** 10.1093/noajnl/vdae058

**Published:** 2024-04-20

**Authors:** Han T N Nguyen, Bailey H Duhon, Hsuan-Chih Kuo, Melanie Fisher, Olivia M Brickey, Lisa Zhang, Jose J Otero, Daniel M Prevedello, Oliver F Adunka, Yin Ren

**Affiliations:** Division of Otology, Neurotology, and Cranial Base Surgery, Department of Otolaryngology—Head and Neck Surgery, The Ohio State University Wexner Medical Center, Columbus, Ohio, USA; Division of Otology, Neurotology, and Cranial Base Surgery, Department of Otolaryngology—Head and Neck Surgery, The Ohio State University Wexner Medical Center, Columbus, Ohio, USA; Division of Otology, Neurotology, and Cranial Base Surgery, Department of Otolaryngology—Head and Neck Surgery, The Ohio State University Wexner Medical Center, Columbus, Ohio, USA; Division of Otology, Neurotology, and Cranial Base Surgery, Department of Otolaryngology—Head and Neck Surgery, The Ohio State University Wexner Medical Center, Columbus, Ohio, USA; Division of Otology, Neurotology, and Cranial Base Surgery, Department of Otolaryngology—Head and Neck Surgery, The Ohio State University Wexner Medical Center, Columbus, Ohio, USA; Division of Otology, Neurotology, and Cranial Base Surgery, Department of Otolaryngology—Head and Neck Surgery, The Ohio State University Wexner Medical Center, Columbus, Ohio, USA; Division of Neuropathology, Department of Pathology, The Ohio State University Wexner Medical Center, Columbus, Ohio, USA; Department of Neurosurgery, The Ohio State University Wexner Medical Center, Columbus, Ohio, USA; Division of Otology, Neurotology, and Cranial Base Surgery, Department of Otolaryngology—Head and Neck Surgery, The Ohio State University Wexner Medical Center, Columbus, Ohio, USA; Division of Otology, Neurotology, and Cranial Base Surgery, Department of Otolaryngology—Head and Neck Surgery, The Ohio State University Wexner Medical Center, Columbus, Ohio, USA

**Keywords:** adherence, extracellular matrix, matrix metalloproteinase, tumor microenvironment, vestibular schwannoma

## Abstract

**Background:**

The progression of vestibular schwannoma (VS) is intricately linked with interactions between schwannoma cells and the extracellular matrix. Surgical resection of VS is associated with substantial risks as tumors are adherent to the brainstem and cranial nerves. We evaluate the role of matrix metalloproteinase 9 (MMP9) in VS and explore its potential as a biomarker to classify adherent VS.

**Methods:**

Transcriptomic analysis of a murine schwannoma allograft model and immunohistochemical analysis of 17 human VS were performed. MMP9 abundance was assessed in mouse and human schwannoma cell lines. Transwell studies were performed to evaluate the effect of MMP9 on schwannoma invasion in vitro. Plasma biomarkers were identified from a multiplexed proteomic analysis in 45 prospective VS patients and validated in primary culture. The therapeutic efficacy of MMP9 inhibition was evaluated in a mouse schwannoma model.

**Results:**

MMP9 was the most highly upregulated protease in mouse schwannomas and was significantly enriched in adherent VS, particularly around tumor vasculature. High levels of MMP9 were found in plasma of patients with adherent VS. MMP9 outperformed clinical and radiographic variables to classify adherent VS with outstanding discriminatory ability. Human schwannoma cells secreted MMP9 in response to TNF-α which promoted cellular invasion and adhesion protein expression in vitro. Lastly, MMP9 inhibition decreased mouse schwannoma growth in vivo.

**Conclusions:**

We identify MMP9 as a preoperative biomarker to classify adherent VS. MMP9 may represent a new therapeutic target in adherent VS associated with poor surgical outcomes that lack other viable treatment options.

**Key Points**

1. Aggressive vestibular schwannoma (VS) adheres to brainstem and has worse outcomes following surgery.
2. MMP9 is highly upregulated in adherent VS.
3. MMP9 enhances schwannoma invasion and adhesion and can be inhibited pharmacologically.

**Importance of the Study**

Vestibular schwannoma (VS), the 4th most prevalent intracranial tumor, causes deafness and brainstem compression and represents the hallmark of *NF2*-related schwannomatosis. Few therapeutic options are available, and surgery remains the only cure. A subset of VS adheres tightly to the brainstem and cranial nerves, complicating surgical dissection and leading to poor clinical outcomes. Understanding the molecular drivers of tumor adherence holds both prognostic and therapeutic potential. Using transcriptomics, proteomics, and primary cultures from patients with sporadic VS, we identify MMP9 as a protease overexpressed in adherent VS. MMP9 represents an excellent biomarker to classify adherent VS with outstanding discriminatory ability. MMP9 promotes schwannoma cell invasion and is inhibited by a drug targeting the eIF4 complex. In vivo inhibition of MMP9 reduced tumor growth in mice. Our study motivates further investigation of MMP9 as a therapeutic target for adjuvant therapy in adherent VS.

The progression of benign and malignant tumors is governed by interactions between tumor cells and their stroma. In vestibular schwannoma (VS), a tumor arising from Schwann cells lining the cochleovestibular cranial nerve, the tumor microenvironment (TME) plays a key role in influencing its natural history.^1,2^ As the 4th most prevalent intracranial neoplasm, VS is also the hallmark tumor of *NF2*-related Schwannomatosis (*NF2*-SWN), a devastating genetic tumor disposition syndrome with no effective cure. The development of aggressive subtypes of VS, such as tumors with significant adherence to the brainstem, depends on the complex interplay between intrinsic schwannoma cells and extrinsic effectors provided by the extracellular matrix (ECM) and cells of the TME.^3,4^ Unlike normal matrix, the schwannoma ECM is a dynamic network that is continuously remodeled, which ultimately shapes its clinical phenotype.

Despite significant morbidities associated with VS, there are no FDA-approved therapies. Small, nongrowing tumors are often observed, while stereotactic radiosurgery is only a viable option for a subset of patients.^5^ Microsurgical resection is typically reserved for aggressive, growing, or large tumors. Surgery is becoming increasingly complex because VS is often extremely adherent to eloquent brainstem structures, the cerebellum, and cranial nerves.^6,7^ The loss of arachnoid planes in areas of peritumoral adhesions poses significant risks to the facial and cochleovestibular nerves during tumor dissection, which could result in permanent facial paralysis and deafness. Other complications such as ischemic or venous pontine strokes, transverse and sigmoid sinus thrombosis, brainstem and cerebellum injury are rare, but neurologically devastating and life-threatening.^8^ Adherent VS is also associated with a higher likelihood of less-than-total resection and tumor re-growth.^9^

Prior studies have identified TME alterations that may play a role in the development of VS, including inflammatory cytokines that induce tumor-associated macrophages toward a pro-tumorigenic phenotype,^3,10^ and increased macrophage infiltration are associated with VS growth.^11^ However, none focused on classifying adherent tumors from nonadherent ones, which have significant surgical implications. Furthermore, there is a lack of understanding of the molecular interplay between schwannoma cells and their ECM, and how this interaction contributes to peritumoral adhesion formation and poor clinical outcomes.

Proteases, enzymes with essential roles in protein degradation and organogenesis, are also critical in tumorigenesis and metastasis.^12^ In malignant and desmoplastic cancers, zinc endopeptidases such as matrix metalloproteinases (MMPs) degrade basement membrane, induce endothelial cell migration in neo-angiogenesis, and facilitate metastasis.^13^ Specifically, MMP2 and MMP9 correlate with disease progression and poor outcomes in multiple cancer types.^13,14^ Although VS is histologically benign, features such as the loss of anatomic separations between the adherent tumor capsule and brainstem in adherent VS resemble that of malignant tumors.^15^ Evidence also suggests that elevated MMP activity, particularly in Antoni B regions, results in increased ECM remodeling and angiogenesis in cystic VS.^16–18^

Leveraging a mouse schwannoma model, a human NF2^−/−^ schwannoma cell line, primary tumor cultures and samples from prospectively enrolled patients undergoing surgery for VS, we characterize the transcriptome of mouse schwannoma and identify MMP9 as a significantly upregulated protease during tumor progression and in adherent human VS. Proteomic analysis of plasma from patients with adherent VS further establishes MMP9 as a biomarker for adherent VS with excellent discriminatory ability. MMP9, produced by schwannoma cells, promotes schwannoma invasion in response to TNF-α and can be inhibited by targeting the eukaryotic translation initiation factor complex, eIF4F. Treatment with MMP9 inhibitors reduced schwannoma growth in mice. Together, this motivates further investigation into MMP9 as a therapeutic target for patients with adherent VS that have poor surgical outcomes and no viable treatments.

## Materials and Methods

Detailed methods are available in Supplementary Materials.

### Patient Samples

Formalin-fixed, paraffin-embedded tumors are obtained retrospectively from the archived tissue bank at Ohio State University. Fresh VS tissue and plasma samples are obtained from prospectively enrolled patients undergoing surgical resection. All patients provided written informed consent following institutional approval (IRB#1994H0241). All diagnoses were confirmed by a neuropathologist per 2016 WHO guidelines.

### Clinical Data

Tumor adherence was independently assessed intraoperatively by multiple surgeons in a semi-quantitative format. Tumor was designated as nonadherent if no adhesion between the tumor capsule and brainstem or cerebellum was noted and the tumor was completely dissected from the brainstem and cranial nerves. The tumor was deemed adherent if: (1) the arachnoid plane between the tumor capsule and posterior fossa structures was lost, (2) sharp dissection of the tumor from the brainstem was required, or (3) if significant adhesions prevented complete dissection of the tumor from neurovasculature and cranial nerves resulting in tumor left in situ.

Cell culture and animal studies.—Animal work was approved by the Institutional Animal Care and Use Committee (#2022A00000043). A mouse NF2^−/−^ schwannoma cell line (gift from Dr. L. Xu, Massachusetts General Hospital) and a human schwannoma cell line from a *NF2*-SWN patient (gift from Dr. M. Giovannini, UCLA) were used. Details on primary VS culture were described.^19^ The sciatic nerve in nude mice was exposed surgically and up to 10 000 tumor cells suspended in Matrigel were slowly injected into the nerve sheath.

### Analysis of Microarray and scRNA-seq Data

Three microarray datasets (GSE141801,^20^ GSE108524,^21^ and GSE39645^22^) were analyzed. Data from sporadic nonirradiated VS were included. Data from scRNA-seq analysis of nonmyelin-forming Schwann cell cluster (nmSC) and myelin-forming Schwann cell cluster (myeSC) were kindly shared by Barret et al. upon request.^23^

### Transwell Invasion Assay

A total of 1 × 10^5^ schwannoma cells was plated on the upper insert in a transwell system coated in rat collagen I. The cells were allowed to migrate after stimulation with TNF-α or MMP9 and quantified by DAPI counterstaining.

### Multiplexed Proteomic Assay

Proteomic profiles of patient plasma (*N* = 6) and age-/gender-matched healthy controls (*N* = 6) were obtained using SOMAscan Assay (SomaLogic, Boulder, CO).

### In Vivo Inhibition of MMP9

Mice bearing subcutaneous MD-MSC tumor allografts were treated with intraperitoneal injections of MMP9-IN-I, silvestrol, or saline and tumor burden was monitored over 2 weeks.

## Results

### Transcriptomic Analysis of Human VS and Mouse Schwannoma Reveals MMP9 as a Highly Upregulated Protease

**Figure 1.**
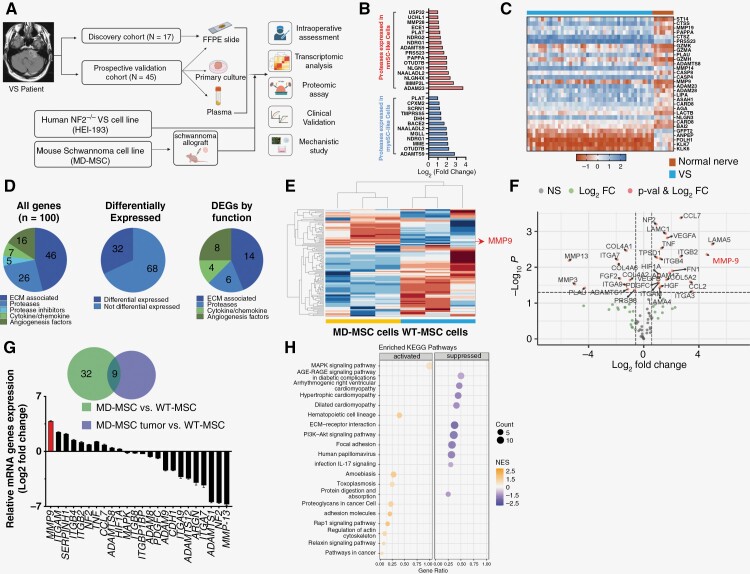
Transcriptomic analyses of human VS and mouse schwannoma identifies MMP9 as a highly upregulated protease. (A) Overview of approach. Tumor and plasma samples are obtained from patients with sporadic VS undergoing surgical resection. A murine schwannoma allograft model is established by implanting NF2^−/−^ Schwann cells into the sciatic nerve sheath. Combining clinical intraoperative assessments, biomarkers are identified and validated. (B) scRNA-seq VS data highlights extracellular proteases enriched in nonmyelinating Schwann cells (nmSC, red) and myelinating Schwann cells (myeSC, blue). (C) Heatmap comparing mRNA expression of top 30 differentially expressed proteases between VS (*n* = 36) and normal nerve (*n* = 7) using published microarray data (GSE141801). (D) Depiction of function of genes profiled in transcriptomic analysis (ECM components, proteases, protease inhibitors, secreted factors and angiogenesis factors) and breakdown of differentially expressed genes. (E) Two-dimensional unsupervised hierarchical clustering of log2 transformed *z*-scored data of MD-MSC and WT-MSCs in vitro. (F) Volcano plot showing significance vs means of differential fold change comparing MD-MSC and WT-MSC. Data is reported as *x*-axis = log_2_ (fold change of MD-MSC vs WT-MSC cells), *y*-axis = −log10 *P*-value, using multi-sample *t* test with FDR correction. *N* = 6 schwannomas were used. (G) Relative mRNA gene expression of 24 DEGs from implanted tumors validated in WT-MSC and MD-MSC cells by qRT-PCR. MMP9 is highlighted in red and represents the most differentially expressed gene. (H) Dot plot showing GO enrichment analysis in schwannomas.

In VS, the complex interplay between tumor cells and their microenvironment influences the highly variable clinical phenotypes and outcomes.^10^ To better understand this interaction, we profiled genes with known roles in ECM remodeling with a focus on proteases, and enzymes with critical roles in the degradation of cancer ECM (Figure 1A). As prior work has shown schwannoma cells transition to a nonmyelinating, injury-like phenotype during tumor progression, we obtained scRNA-seq data from 15 sporadic VS and queried the expression of proteases in myelinating (myeSC) and nonmyelinating clusters (nmSC).^23^ Some nmSC display a repair- and injury-like phenotype, demonstrating an inflammatory transcriptomic profile and actively recruiting monocytes as compared to the quiescent nature of mySC.^23^ We cross-referenced differentially expressed genes (DEGs) with *MEROPS*, a database of over 1000 human proteases and homologs (https://www.ebi.ac.uk/merops/). There was a significant enrichment of metallo- and serine proteases (*ADAM23, ADAMTS9, PAPPA, ECE1, MMP28*, *PRSS23*) in nmSC (Supplementary Table 1), suggesting that nmSCs may influence ECM remodeling by protease activation (Figure 1B).

Given the rarity of VS, we next validated these findings in larger VS cohorts. Nonetheless, limited data was available on the degree of tumor adherence. We obtained 3 publicly available RNA microarray datasets and identified up to 30 differentially expressed proteases (Supplementary Table 2). In the largest dataset (GSE141801) comprised of 49 VS (36 sporadic, 13 *NF2*-SWN), differentially abundant proteases included metalloproteases *(MMP9, MMP14, MMP19),* cathepsins (*CTSS, CTSZ),* and granzymes *(GZMA, GZMK,* and *GZMH,*Figure 1C).^20^ Similar findings were seen in 2 additional datasets^21,22^ (Supplementary Figure 1A and B), suggesting that despite diverse tumor and patient origins, there was a common set of dysregulated proteases in VS.

To further investigate the expression of proteases in VS, we employed a murine Nf2^−/−^ schwannoma cell line (MD-MSC) and implanted them into the sciatic nerve of nude mice to establish tumor allografts. We performed quantitative multiplexed transcriptomic profiling of 100 genes involved in collagen synthesis, cell–cell adhesion, angiogenesis, immune cell recruitment, cytokines/chemokines, proteases and inhibitors in implanted schwannoma tumors, MD-MSC cells, and wild-type Schwann cells (WT-MSC) (Supplementary Table 3; Supplementary Figure 1C). We were interested in defining genes that were dysregulated in implanted schwannoma tumors compared to wild-type Schwann cells, but whose expression was not influenced by the in vivo vs in vitro experimental conditions (Figure 1D). Two-dimensional unsupervised hierarchical clustering of MD-MSC and WT-MSCs demonstrated 2 clusters (Figure 1E). Forty-eight genes were differentially expressed: 32 between MD-MSC and WT-MSCs, 24 comparing implanted tumors and WT-MSCs, and 9 DEGs common to both comparisons (Supplementary Table 4). There was an enrichment of metalloproteases (*MMP9, MMP13, ADAMTS1*), integrins (*ITGA7/9, ITGB2*), collagens (*COL4A2, COL5A2*) and HGF (*P* < .05). MMP9 was the most abundantly differentially expressed protease (Figure 1F). We then validated the 24 DEGs (12 upregulated and 12 downregulated genes) between tumors and WT-MSCs. Seventeen (71%, 8 upregulated and 9 downregulated) remained concordantly differentially expressed in MD-MSC cells compared to WT-MSC cells in vitro. Of 32 DEGs between MD-MSC and WT-MSC, 25 (80%, 15 upregulated and 10 downregulated) remained concordantly differentially expressed. Our results confirmed *MMP-9, ITGAM, ITGB4, TNF, MMP-13,* and *ADAMTS1* as intrinsic markers of MD-MSCs, validating their differential expression as reflecting actual biological differences and highlighting the distinct nature of MD-MSC cells. Notably, MMP-9 was the most enriched gene in MD-MSC cells and overexpressed by over 15-fold (Figure 1G). Gene ontology (GO) term enrichment analysis demonstrated enrichment in MAPK and cell–cell adhesion pathways, whereas PI3K-Akt signaling and ECM-receptor interactions were suppressed (Figure 1H, Supplementary Figure 1D–E**).** Together, these findings suggest that VS upregulate programs in collagen synthesis, integrin expression, and proteases including MMP9.

### Increased MMP9 During Progression of Mouse Schwannomas and Human VS

**Figure 2.**
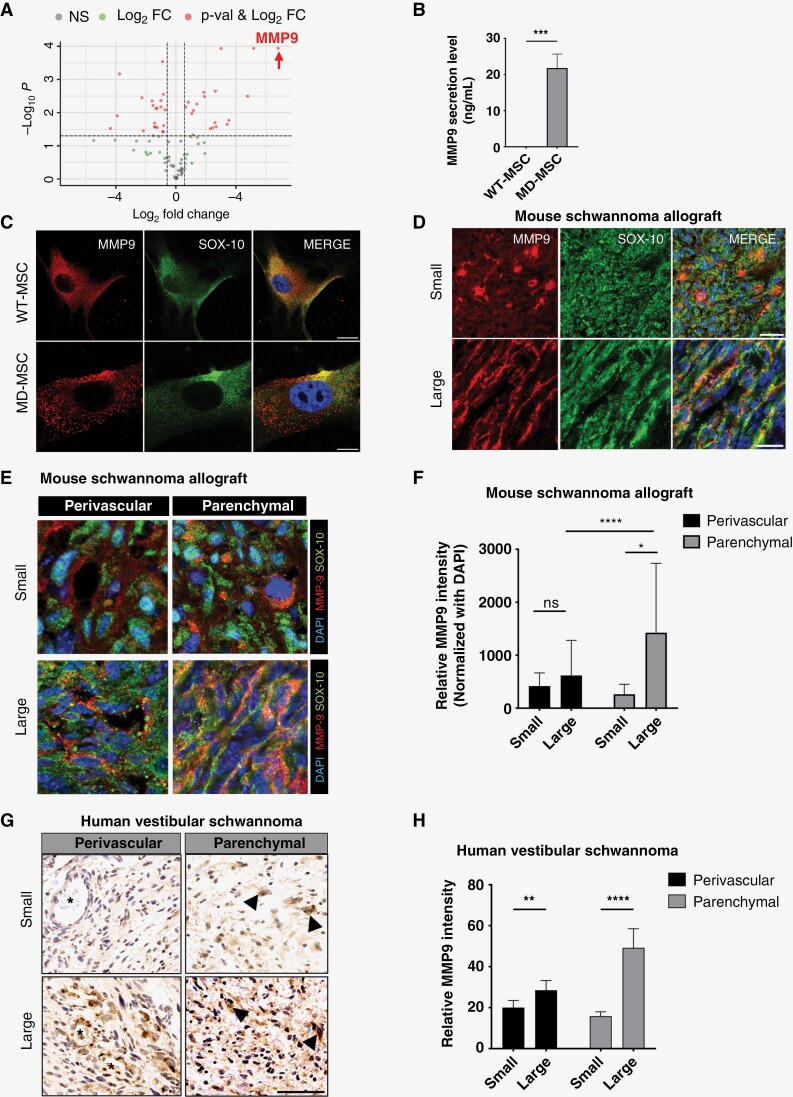
MMP9 is secreted by schwannoma cells and expressed in the tumor parenchyma. (A) Volcano plot of differentially abundant genes in ECM remodeling in mouse schwannoma allograft or WT-MSCs showing MMP9 elevation, using multi-sample *t* test with FDR correction. (B) MMP9 is secreted by mouse schwannoma cells but not WT-MSC. *N* = 6 biologically independent samples, ***P <* *.002 by 2-sided Mann–Whitney *U*-test. (C) Representative confocal immunofluorescence of MMP9 expression in WT-MSC (top) and MD-MSC (bottom). MMP9, SOX-10 are presented in red and green pseudo-colors respectively. Scale bar, 100 µm. (D) Representative images of MMP9 expression (red) in mouse schwannomas (SOX-10, green) at different stages of tumor progression (top: small, *N* = 3; bottom: large, *N* = 4). Small tumor was defined as tumors harvested at 1 week after implantation (average size = 5 mm) and large tumor was defined as tumors at 3 weeks after implantation (average size = 10 mm). Scale bar, 100 µm. (E) Representative images of MMP9 expression in perivascular and parenchymal region at different stages of mouse tumor progression (DAPI, blue; MMP9, red; SOX-10, green). (F) Quantification of MMP-9 intensity from E. Bars represent mean ± SD, *P < .05, ****P < .0001 by 2-way ANOVA with Bonferroni–Dunn post hoc analysis. Scale bar, 50 µm. (G) Representative IHC images and (H) quantification of MMP9 staining intensity in human VS. Bars represent mean ± SD, **P = .0019, ****P < .0001 by 2-way ANOVA with Bonferroni–Dunn post hoc analysis.

Accumulating evidence suggests that MMP9 is secreted by both tumor and stromal cells which promotes tumor growth and angiogenesis.^24,25^ In murine schwannoma allografts, *MMP9* expression was significantly enriched compared to WT-MSCs (log_2_fold change = 6.8, *P* < .05, Figure 2A). Additionally, MD-MSC cells secreted more than 20-fold higher levels of MMP9 than WT-MSCs (Figure 2B). WT-MSCs exhibited a cytoplasmic pattern of MMP9 expression, whereas MD-MSCs displayed a more intense, membranous, and extracellular pattern (Figure 2C). MMP9 is synthesized as a preproenzyme, secreted extracellularly as an inactive zymogen and activated by other proteases.^26^ Since the MMP9 antibody does not fully distinguish active MMP9 from its precursor, the different subcellular localization may be due to differences in cleaved and activated MMP9.^27,28^ Since tissue inhibitors of metalloproteases (TIMPs) are endogenous inhibitors of MMPs, we performed qRT-PCR analysis of *TIMP*-*1* to *4. TIMP-*3*/4* were significantly downregulated, suggesting MMP9 may be partly regulated by their endogenous inhibitors (Supplementary Figure 2A).

To examine the spatial expression of MMP9 during mouse schwannoma progression, we stained tumors 1 week after implantation (small) and 3 weeks after implantation when tumors reached 1 cm in diameter (large). MMP9 initially concentrated around SOX-10 + schwannoma cells. As the tumor grew, MMP9 expression was more widespread (Figure 2D). MMP9 expression within the tumor parenchyma increased by 5-fold in large tumors (*P* = .028, Figure 2E and F). MMP9 expression in the perivascular region also increased but to a lesser extent. This pattern of MMP9 staining is similar to other disease conditions where proteases mediate disease progression.^29^ Analogous to the mouse model, we observed a similar pattern of MMP9 expression in human VS (Figure 2G). There was a moderate increase in perivascular MMP9 in large compared to small VS (average tumor diameter 3.9 vs 1.1 cm, *P* = .0019), and a significant increase in parenchymal MMP9 in large tumors (Figure 2H, *P* < .0001).

### MMP9 Is Enriched in Adherent Human Vestibular Schwannoma

**Figure 3.**
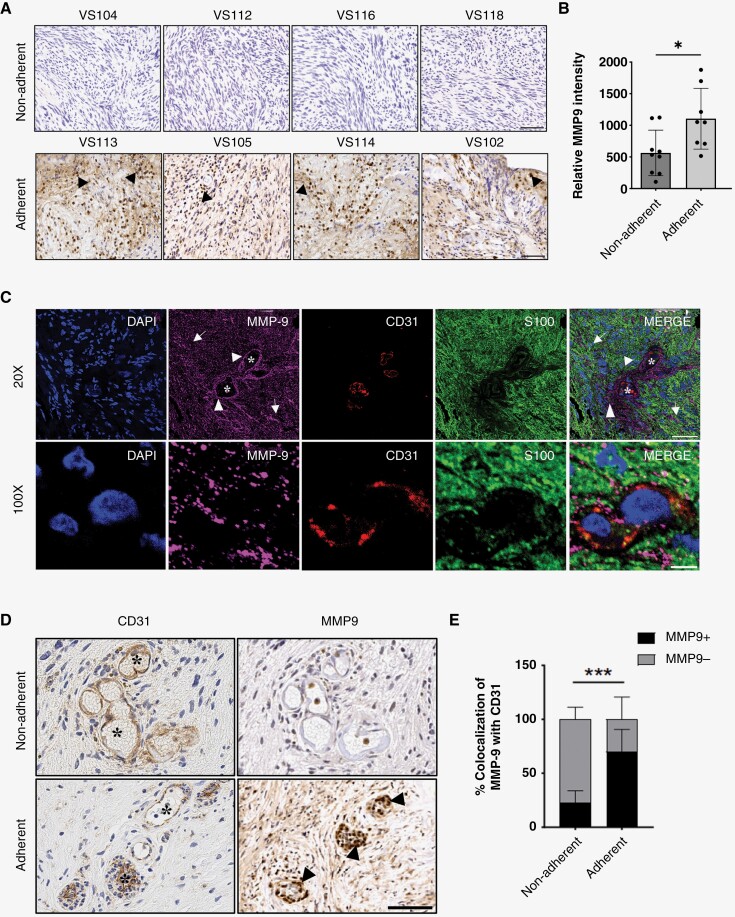
MMP9 is overexpressed in patients with adherent VS. (A) Immunohistochemical staining of MMP9 in VS tissue. Top row, representative images from *N* = 9 patients with nonadherent tumors. Bottom row, representative images from *N* = 8 tumors adherent to the brainstem. MMP9 expression is indicated by arrowheads. Scale bar, 100 µm. (B) Quantification of MMP9 expression in adherent and nonadherent VS. For each patient, 10–15 representative ROIs were chosen. Bars represent mean ± SD, *P < .05 by 2-sided Mann–Whitney *U*-test. (C) Representative immunofluorescence images showing colocalization of MMP9 with CD31 and S100 in VS. Representative low-powered fields (top row, scale bar is 100 µm) and high-powered fields (100×, bottom row, scale bar is 50 µm) are shown. DAPI, blue; MMP9, purple; CD31, red; S100, green. Blood vessels are indicated by *, parenchymal MMP9 by arrows, and perivascular MMP9 by arrowheads. (D) Representative IHC images of serial sections with CD31 or MMP9 staining in nonadherent (top) and adherent VS (bottom). Scale bar, 50 μm. Blood vessels indicated by *, MMP9 by filled arrowheads. (E) Quantification of CD31 + vessels with colocalized MMP9 (black bars) expression. Mean ± SD are shown. *N* = 10 tumors were used for quantification with 10–12 ROIs from each tumor, ***P < .001 by 2-way ANOVA with Bonferroni–Dunn post hoc analysis.

MMP9-producing leukocytes promote neo-angiogenesis^30^ via the upregulation of vascular endothelial growth factor (VEGF) secretion and disruption of the blood-brain barrier.^31^ Furthermore, MMP9 degrades type IV and V collagen which disrupts vascular permeability and facilitates cellular migration during metastasis.^32^ We hypothesized that MMP9 in VS may enhance vascular permeability, break down arachnoid planes, and facilitate the outgrowth of schwannoma cells onto the brainstem, thereby making the tumor more adherent. With increased perivascular and parenchymal expression of MMP9 in large compared to small VS, we next sought to determine whether MMP9 expression was enriched in adherent VS. In a small retrospective cohort of 17 VS patients, adherent tumors stained strongly positive for MMP9 and largely localized to the extracellular milieu (Figure 3A and B, Table 1). MMP9 was strongly colocalized with CD31/PECAM1 + endothelial cells and S100B + schwannoma cells (Figure 3C and Supplementary Figure 2B). To specifically compare MMP9 expression around the tumor vasculature in adherent and nonadherent tumors, we identified regions containing CD31 + vessels and queried the colocalization of MMP9 expression on consecutive histological sections (Figure 3D). In nonadherent VS, 30% CD31 + vessels colocalized with MMP9. By contrast, 70% of adherent VS endothelium also showed perivascular MMP9 expression (Figure 3E). Together, our data indicates adherent VS are enriched in MMP9, and there was a higher number of blood vessels that colocalized with MMP9 expression.

**Table 1.** Demographics of the retrospective patient cohort.

ID	Age	Gender	Tumor diameter (cm)	Tumor volume (cm^3^)	PTA (dB HL)	WRS (%)	Adherent tumor	Tumor growth	Brainstem compression	Gross total resection
VS104	61	M	1.9	16.6	35	92	No	No	Yes	Yes
VS108	76	M	1.7	3.5	41	60	No	Yes	Yes	Yes
VS110	66	M	1.3	1.4	33	92	No	Yes	Yes	Yes
VS112	53	M	2.2	29.0	29	18	No	N/A	Yes	Yes
VS115	36	M	0.9	2.4	13	100	No	Yes	No	Yes
VS116	60	M	1.8	19.3	25	88	No	No	Yes	Yes
VS117	75	F	2	7.5	37	24	No	Yes	Yes	Yes
VS118	73	M	1.9	16.7	66	0	No	No	Yes	No
VS122	75	F	1	6.7	48	36	No	Yes	No	Yes
VS102	31	M	4.2	247.4	110	0	Yes	N/A	Yes	No
VS103	34	M	1.9	18.9	50	100	Yes	No	Yes	Yes
VS105	81	F	2.3	47.6	53	64	Yes	Yes	Yes	No
VS107	60	F	1.2	12.4	81	0	Yes	No	No	Yes
VS113	56	M	2	22.8	41	92	Yes	No	Yes	Yes
VS114	37	M	3.4	117.5	51	72	Yes	No	Yes	No
VS119	59	F	2.2	28.2	22	100	Yes	N/A	Yes	Yes
VS121	33	M	3.7	148.8	46	88	Yes	No	Yes	Yes

Abbreviation: PTA, pure tone average; WRS, word recognition score.

### MMP9 Is a Classifying Biomarker for Adherent VS

**Figure 4.**
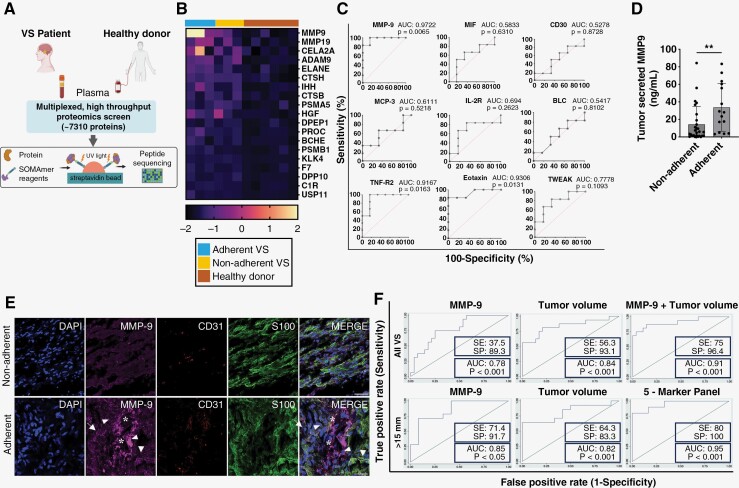
MMP9 is a tumor classifying biomarker for adherent VS. (A) Illustration of workflow. Plasma from VS patients and healthy donors were collected and analyzed using a high-throughput multiplexed proteomic analysis platform. (B) Heatmap showing the most differentially expressed proteases in adherent VS (*N* = 3), nonadherent VS (*N* = 3), and healthy donors matched in age and gender (*N* = 6). (C) Receiver-operating characteristic curve (ROC) analysis of 9 plasma biomarkers from the proteomics assay in classifying VS from healthy donors. AUC, area under the curve. (D) In primary tumor cultures, MMP9 is significantly elevated in secretions from adherent VS (*N* = 16, mean 49.7 ng/ml) compared to nonadherent VS (*N* = 29, mean 13.5 ng/ml). Values are mean ± SD, **P = .014 by 2-sided Mann–Whitney *U*-test. (E) Representative multi-color immunofluorescence images showing colocalization of MMP9 with CD31 and S100 in VS from the prospective cohort. Scale bar is 100 µm. DAPI, blue; MMP9, purple; CD31, red; S100, green. Blood vessels are indicated by *, parenchymal MMP9 by arrows, and perivascular MMP9 by arrowheads. (F) ROC analysis comparing various clinical and radiographic variables to classify adherent from nonadherent VS. The 5-marker panel included: MMP9, tumor volume, brainstem compression, CSF cleft and cystic change. SE, sensitivity, SP, specificity.

While the complex decision to perform operative intervention is based on an array of clinical factors, there is a lack of preoperative biomarkers to predict the intraoperative experience and correlate with postoperative outcomes.^33^ We therefore examined the potential of MMP9 to classify VS that are adherent and portend poor outcomes. We utilized a high-throughput proteomic platform using aptamers to simultaneously screen 7310 plasma proteins. Each biotinylated aptamer is modified with a photocleavable linker and fluorescent tag. Upon protein binding, the complex is captured by streptavidin and its abundance is quantified by fluorescence (Figure 4A). Six VS patients, 3 with adherent tumors and 3 with nonadherent VS, were age- and gender-matched to 6 healthy donors (Supplementary Table 6). Focusing on proteases, we identified an over-abundance of MMP9, MMP19, ADAM9, cathepsins, a serine protease (DPP10), HGF, and KLK4 in VS (all *p*-values adjusted for multiple comparisons < .05, Figure 4B and Supplementary Table 7). A recent study identified 9 plasma biomarkers that discriminated VS from healthy controls,^34^ with TNF-R2, MIF, and CD30 as the top 3. Comparative receiver-operating characteristic curve (ROC) analyses showed MMP9 performed favorably (AUC = 0.97, *P* = .007), while TNF-R2 (AUC = 0.9197, *P* = .016) and eotaxin (AUC = 0.9306, *P* = .013) remained significant (Figure 4C).

To explore whether MMP9 could distinguish adherent from nonadherent tumors in an independent contemporary cohort, we prospectively enrolled 45 VS patients and established primary tumor cultures (Supplementary Figure 4A). This prospective cohort allowed for a more objective assessment of tumor adherence, as the operator experience was standardized, and degree of tumor adherence was quantified intraoperatively and independently. Twenty-nine had nonadherent VS and 16 had adherent tumors (Table 2). Adherent VS primary cultures secreted higher levels of MMP9 than nonadherent VS (49.7 vs 13.8 ng/ml, *P* = .014) (Figure 4D). While adherent tumors may be larger than nonadherent tumors overall, in a size-matched sub-analysis of adherent and nonadherent tumors (*n* = 5 per group, tumor diameter 2.7 vs 2.7 cm), adherent VS still secreted higher levels of MMP9 (87.4 vs 2.6 ng/ml, *P* = .012) (Supplementary Table 5). Immunofluorescence staining also showed that MMP9 expression was enriched in adherent tumors in this cohort (Figure 4E). ROC analysis identified both MMP9 and tumor volume as classifying biomarkers (Figure 4F, Supplementary Figure 4, and Supplementary Table 8). Nonetheless, MMP9 alone may not be a perfect predictor of adherence, as larger tumors often can be more adherent. When MMP9 was combined with tumor volume, multivariable logistic regression identified the combination as an excellent biomarker (AUC_MMP9+vol_ = 0.91, Sensitivity 75%, Specificity 96%, Figure 4F). However, the 2 biomarkers are independent, as tumor volume did not correlate with MMP9 in plasma or in tumor secretion. Moreover, several large nonadherent tumors expressed low levels of MMP9 while small adherent tumors showed elevated MMP9 (Supplementary Figure 3).

**Table 2.** Comparison of adherent and nonadherent VS in the prospective validation cohort.

Validation cohort (*n* = 45)			
	Adherent VS (*n* = 16)	Nonadherent VS (*n* = 29)	*P*-value
Demographics			
Age, years ± SD	51.8 ± 13	55.3 ± 11	.36
Sex			.19
Male, *n* (%)	9 (56%)	11 (38%)	
Female, *n* (%)	7 (44%)	18 (62%)	
Tumor characteristics			
Tumor volume, cm^3^ ± SD	127.8 ± 12.1	16.4 ± 2.4	**.002**
Cystic, *n* (%)	4 (25%)	0	**.012**
CSF cleft, *n* (%)	8 (50%)	20 (69%)	.20
Brainstem compression, *n* (%)	12 (75%)	7 (32%)	**.001**
Hearing status			
PTA, dB HL	49.0	45.1	.65
WRS, %	54.7	61.8	.55
Extent of resection			**<.001**
Gross/near-total, *n* (%)	5 (31%)	28 (97%)	
Sub-total, *n* (%)	11 (69%)	1 (3%)	

We next investigated the predictive potential of MMP9 in medium-to-large tumors (maximum diameter >15 mm). MMP9 was the best performing individual biomarker (AUC = 0.85) followed by tumor volume. The 5-marker panel (MMP9, tumor volume, presence of brainstem compression, CSF cleft, and cystic change) exhibited the best performance (AUC_5-marker_ = 0.95, Sensitivity 80%, Specificity 100%) (Figure 4F and Supplementary Table 8). Taken together, our results underscore the potential of MMP9 as a clinical marker independent of tumor size that may help lay the foundation for preoperative classification of adherent VS.

### MMP9 Expression Is Regulated by the eIF4 Complex and Promotes Schwannoma Cell Adhesion and Invasion In Vitro

**Figure 5.**
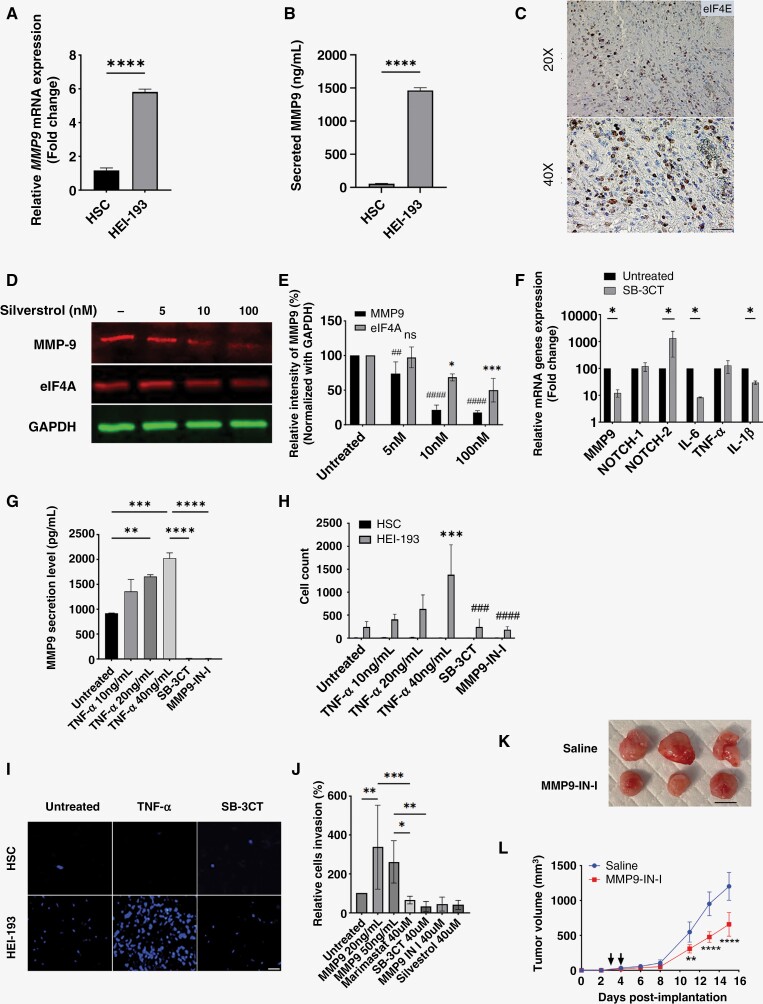
MMP9 expression is regulated by components of the eIF4F complex and enhances schwannoma cell invasion through collagen in vitro. (A) *MMP9* mRNA expression is highly abundant in HEI-193 cells compared to Schwann cells (HSC). Mean ± SD of 3 biologically independent samples, ****P < .0001 by 2-sided Mann–Whitney *U*-test. (B) MMP9 is enriched in conditioned media from HEI-193 cells. Mean ± SD of *N* = 6 biologically independent samples are shown, ****P < .0001 by 2-sided Mann–Whitney *U*-test. (C) Representative image from IHC staining shows overexpression of eIF4E in sporadic VS. Scale bar, 50 µm. (D) MMP9 and eIF4A protein expression are inhibited by silvestrol. (E) Quantification of band intensities from 3 independent experiments are shown as mean ± SD. ##*P* < .002, ####*P* < .0001 MMP9 vs untreated; *P < .05, ****P < .0001 eIF4A vs untreated by one-way ANOVA with post hoc Tukey test. (F) Inhibition of MMP9 in HEI-193 using a small molecule inhibitor (SB-CT) resulted in alterations in *NOTCH-2, IL-6,* and *IL-1β*. *MMP9* and *IL-6* mRNA expression was reduced by 10-fold and IL-1β by 8-fold, whereas NOTCH-2 was increased by 10-fold. Mean ± SD of *N* = 3 independent samples are shown, *P < .05 by 2-way ANOVA with Bonferroni–Dunn post hoc analysis. (G) MMP9 secretion by HEI-193 is enhanced with TNF-α stimulation and abrogated following treatment with MMP9 inhibitors. Data from 3 independent experiments are shown as mean ± SD.*P < .05, **P < .002 ***P < .0002, ****P < .0001 by one way ANOVA with post hoc Tukey test. (H) Transwell assay to assess invasion of schwannoma cells through collagen-I in the presence of TNF-α or MMP9 inhibitors. TNF-α significantly enhanced the number of invading HEI-193 cells by over 100-fold but did not affect HSCs. Data from 3 independent experiments are shown as mean ± SD. *P < .05, ****P < .0001 vs untreated; ###*P* < .0002, ####*P* < .0001 vs SB-3CT and MMP9-IN-I, one way ANOVA with post hoc Tukey test. (I) Representative immunofluorescence images from transwell assays. Six biologically independent experiments were performed. Scale bar, 100 µm. (J) Recombinant MMP9 enhances HEI-193 invasion and reproduced the behavior seen from TNF-α stimulation. Cellular invasion was enhanced with MMP9 and inhibited by Marimastat, SB-3CT, MMP9-IN-I and silvestrol. Data from 3 independent experiments are shown as mean ± SD. *P < .05, **P < .002, ***P < .0002, ****P < .0001 by one way ANOVA with post hoc Tukey test. (K) Representative images of tumor allografts from saline controls and MMP9-IN-I treated animals. (L) Tumor growth of MMP9-IN-I treated and saline control mice bearing subcutaneous schwannoma allografts (*N* = 5 each). Black arrowheads indicate treatments on days 3 and 4. **P = .0024, ****P < .0001 by 2-way ANOVA with Bonferroni–Dunn post hoc analysis.

After establishing MMP9’s role as a potential biomarker of adherent VS, we next sought to provide mechanistic insight into the function of tumor-associated MMP9 and its regulation. MMP inhibitors have thus far underperformed in therapeutic studies, partly owing to the presence of multitudes of MMP enhancers and activators.^35^ The NF2^−/−^ human schwannoma cell line (HEI-193) overexpress and secrete MMP9 in vitro (Figure 5A and B). Previous work has shown recruitment of the eukaryotic initiation factor 4F (eIF4F subunit specifically) to the 5ʹ UTR of oncogenic kinases and leads to constitutive kinase activation and translation of *MMP9*.^36–38^ Labeling of eIF4E, an integral cap-binding protein subunit of eIF4, in VS tissues showed expression in the cytoplasm and nucleus, consistent with its role in translation initiation and mRNA export (Figure 5C). To investigate whether the eIF4 complex regulates MMP9 expression, we treated HEI-193 cells with silvestrol, a small molecule targeting eIF4A1/2 helicase that potently induced cell cycle arrest.^38^ Inhibition of eIF4 complex by silvestrol at 10 nM was associated with over 80% suppression of MMP9 (Figure 5D and E and Supplementary Figure 5A and B).

We next investigated the mechanism by which MMP9 activity could mediate schwannoma cell invasion and increased cellular adhesion. NOTCH signaling has been shown to have both oncogenic and tumor suppressor properties in cancer.^39–41^ In solid tumors, inhibition of NOTCH-2 has been associated with an increase in cellular invasion, driven by elevated MMP activity and enhanced secretion of IL-6 and IL-1β.^40,42^ However, the role of MMP9 in modulating NOTCH activity in VS is not well explored. Inhibition of MMP9 by a small molecule inhibitor (SB-3CT) increased *NOTCH-2* by over 10-fold, whereas *IL-6* and *IL-1β* expressions were reduced (Figure 5F). TNF-α is secreted by VS cells in patients with poor hearing,^43^ and incubation with TNF-α led to a dose-dependent upregulation of MMP9 secretion (Figure 5G). Furthermore, TNF-α also led to upregulation of αvβ3 integrin and a 3-fold increase in expression of the cell–cell adhesion molecule N-cadherin in HEI-193 cells (Supplementary Figure 5C and D), 2 cell-surface proteins whose overexpression is observed in metastatic cancers.^44,45^

We next examined the effect of MMP9 on schwannoma cell invasion in vitro. TNF-α stimulation of schwannoma cells increased the number of invading cells by over 100-fold without affecting viability (*P* < .0002) (Figure 5H and I, Supplementary Figure 5E and 6A) compared to Schwann cells. The effect of TNF-α stimulation was inhibited by subsequent addition of MMP9 inhibitors (SB-3CT or MMP9-IN-I) or silvestrol (Figure 5H and Supplementary Figure 6F and -G).^46,47^ As a positive control, HEI-193 cells invaded through collagen-I with recombinant MMP9, an effect that was inhibited with MMP inhibitors and silvestrol (Figure 5J). Similar results were observed in MD-MSCs (Supplementary Figure 6).

Finally, to assess the therapeutic potential of MMP9 inhibitors in vivo, mice bearing subcutaneous schwannoma allografts were treated with silvestrol (1.5 mg/kg or 3 mg/kg) or MMP9-IN-I (3 mg/kg). We observed a significant reduction of tumor burden by 50% in MMP9-IN-I treated animals (Figure 5K and L). By contrast, silvestrol treatment was less well-tolerated and exhibited signs of toxicity evidenced by significant weight loss (Supplementary Figure 7). Together, our data provides mechanistic insight into the function of MMP9 in enhancing schwannoma cell invasion and adhesion, identifies eIF4 complex as a regulator of MMP9, and provides proof-of-concept data on the therapeutic efficacy of inhibiting MMP9.

## Discussion

Focusing on the role of ECM remodeling in VS progression, we demonstrate that MMP9 is an extracellular protease overexpressed in a mouse schwannoma model and in patients with adherent VS. In a pro-inflammatory TME, MMP9 mediates the development of adherent VS through upregulation of cell–cell adhesion molecules and increased cell invasion. Inhibition of MMP9- may represent a novel therapeutic approach in VS.

During cancer progression, the continuous replacement of normal ECM is influenced by a dynamic array of infiltrating immune cells, cytokines, and enzymes such as proteases which remodel the ECM structure and composition to promote tumor migration.^12^ The progression of VS is accompanied by a concomitant ECM remodeling, which results in structural and biochemical alterations both within the tumor and its microenvironment.^48^ This complex and dynamic process, influenced by the interplay between schwannoma cells and stroma, contributes to the variable natural history and symptoms. Many VS patients undergo surgery due to the presence of severe symptoms or evidence of brainstem compression, with the hope of preventing additional complications.^5^ However, there are no consensus staging or grading systems to classify VS and guide surgical decision-making. A clinically useful biomarker should identify patients at high risk for postoperative complications and therefore help make more data-driven decisions. As more VS are being observed or undergo radiation, tumors that undergo surgery are typically larger, cystic, and more aggressive, making surgery increasingly complex.^5^ Moreover, surgeons are tasked with deciding between complete tumor removal and preservation of neurological function. During resection, VS that are adherent from the brainstem or cranial nerves are difficult, if not impossible, to completely remove.^49^ There are currently few tools to determine the degree of tumor adherence *prior to* surgery that also correlate with postoperative outcomes.^50^ The value of MMP9 as a biomarker lies in its ability to aid in predicting the intraoperative experience and postoperative outcomes. In our cohort, adherent tumors contained high levels of MMP9, and elevated circulating MMP9 in VS may reflect ongoing ECM remodeling resulting in “leakage” of tumor-secreted proteins.^16,51^ MMP9 is an independent factor in predicting the development of adherent tumors apart from tumor size, and our data provides an additional tool for preoperative planning, predicting the intraoperative difficulty of tumor dissection, and patient counseling.

Most existing studies on VS are small retrospective series with limited generalizability. To overcome these challenges, we analyzed data from 3 independent cohorts consisting of almost 200 patients and combined it with single-cell transcriptomic analysis from 15 VS patients.^23^ We focused on extracellular peptidases for several reasons. First, proteases are central to cancer hallmarks including neo-angiogenesis, recruitment of immune cells, and enhancement of cellular invasion,^52^ many akin to the development of adherent VS.^13^ Second, previous work has implicated MMPs, caspases, and kallikreins in VS patients with poor hearing or those that underwent subtotal resections.^18^ Third, proteases serve as excellent biomarkers due to their catalytic activity, enabling highly sensitive measurements in complex samples. In addition to MMPs, other dysregulated proteases included *ADAM28, ADAM23, PRSS23,* and *PAPPA*. ADAMs could affect cell migration and angiogenesis but their role in VS progression is yet to be defined.^53^ PAPPA cleaves insulin-like growth factor-binding proteins to release insulin-like growth factors to promote tumor proliferation and survival.^54^ Future studies should continue to expand and investigate the protease repertoire in VS.

The challenges posed by the inability to achieve gross total resection of adherent VS and elevated postoperative morbidities underscore the need for an improved understanding of tumor progression. Our data suggests that schwannoma cells are one of the sources of MMP9, and schwannoma cells invade through collagen I due to MMP9-mediated collagen breakdown in vitro. MMP9 also interacts with the tumor matrix via cleaving laminin and aggrecan and ultimately increases the bioavailability of vascular endothelial growth factor (VEGF) during the angiogenic switch.^13^ This is consistent with our findings of enriched MMP9 expression in both perivascular regions and tumor parenchyma. Inhibition of MMP9 led to upregulated *NOTCH-2* and downregulated *IL-6* expression. NOTCH-2 signaling has been shown to regulate endothelial cells by activating specific MMPs, and its inhibition resulted in MMP9 upregulation and activation of PI3K-Akt signaling in gastric cancer.^40^ Therefore, it is possible that MMP9 overactivity may result in reduced NOTCH-2 activity, which subsequently enhances the invasive potential of tumor cells through PI3K/Akt.

Moreover, inflammation-mediated expression of adhesion molecules such as integrins and cadherins may contribute to the development of “sticky” schwannomas. Overexpression of both N-cadherin, a cell–cell adhesion molecule, and αvβ3, an integrin, has been shown to increase cell motility and invasion in aggressive cancers.^55,56^ Specifically, upregulation of αvβ3 in vitro increased FAK signaling and epithelial–mesenchymal transmission of breast cancer cells.^57^ Moreover, αvβ3 is a negative prognostic factor in gliomas possibly due to αvβ3 and MMP colocalization at the site of tissue invasion, highlighting the role of MMP activity in glioma metastasis.^45,58^ Furthermore, N-cadherin upregulation in cholangiocarcinoma increased cellular invasion and is associated with decreased survival.^59^ When N-cadherin is inhibited in melanoma cells, there is a decrease in MMP expression and cellular invasion.^57^ N-cadherin is also involved in priming the premetastatic niche in vitro.^60^ These findings are corroborated in breast cancer, as N-cadherin expression correlated with tumor metastasis, lymph node involvement, and disease recurrence.^61^ While VS is benign, expression of N-cadherin and αvβ3 could facilitate the development of cell adhesions between schwannoma and surrounding tissue, possibly through interactions with MMPs. In fibroblasts and macrophages, IL-1/-2/-6 and TNF-⍺ can all induce MMP2 and MMP9 expression via MAPK.^62^ Many of the DEGs in our model were also linked to MAPK signaling, which has well-known roles in regulating tumor differentiation and tissue invasion.^63^ Taken together, our results provide several mechanistic insights into how MMP9 activity could mediate adherent VS development.

Currently, no FDA-approved pharmacotherapies exist for sporadic VS. The most successful chemotherapeutic, bevacizumab, is effective in 50% of *NF2*-SWN patients and has significant side effects.^64^ While MMP9 represents an attractive therapeutic target, direct inhibitors of MMP9 have met with failures in cancer clinical trials^35^ likely due to MMP9’s pleotropic role in physiologic processes. We observed that silvestrol, an inhibitor of eIF4A1/2 helicases that prevents incorporation of eIF4A into eIF4 complex, effectively inhibited schwannoma invasion in vitro but was toxic in vivo. Our in vivo data in a subcutaneous schwannoma mouse model suggests that a small molecule MMP9 inhibitor demonstrated significant anti-tumor effects and was well tolerated. Therefore, future work should focus on validation of therapeutics targeting the TNF-⍺/MMP9 axis.

The availability of accurate preoperative classification biomarkers to help risk-stratify and predict the intraoperative experience would allow surgeons to better prepare for surgical resection. TNF receptor 2 (TNF-R2), which is involved in activating myeloid-derived suppressor cells, was identified as a plasma biomarker of VS, with IL-16 and S100B also correlating with tumor volume.^33,34^ However, tumor volume is not always correlated with outcomes^65^; as large VS that are soft can be completely resected while small adherent tumors may be difficult to remove completely. Our study focuses on identifying molecular drivers of ECM remodeling and therefore did not focus on tumor volume markers such as S100B.^34^ Nonetheless, the combined biomarker of MMP9 and volume had excellent discriminatory ability, and incorporation of radiographic factors such as the presence of CSF cleft and brainstem compression further improved the performance of the panel. Together, supported by *in silico*, in vivo, and primary culture data, our findings suggest that MMP9 can serve as a biomarker to classify adherent from nonadherent VS.

This study has several limitations. First, the relatively small number of candidate genes in ECM remodeling in our transcriptomic analysis could affect the generalizability of our findings. Second, although tumor adherence was determined in a standardized semi-quantitative format, observer biases could still exist. Future studies to delineate the degree of tumor adherence using quantitative imaging modalities should be considered.^50^ Third, although our in vitro and in vivo findings were generated using established NF2^−/−^ cell lines, they may not accurately recapitulate the phenotype of VS. Additional work utilizing primary VS cultures is needed to confirm our findings. Finally, the small sample size used to analyze MMP9’s performance as a biomarker limits its generalizability.

In summary, using a mouse schwannoma model and VS from patients, we identify MMP9 as a highly active protease during schwannoma growth and in adherent VS, representing an excellent classifying biomarker to distinguish adherent from nonadherent tumors. MMP9 promotes schwannoma invasion and increases expression of cell adhesion molecules and can be targeted by a small molecule inhibitor in vivo. Having the knowledge of tumor MMP9 levels preoperatively could improve patient counseling by predicting intraoperative tumor adherence. In the future, development of technologies to detect MMP9 and therapeutics against MMP9 in adherent VS could represent a breakthrough in the management of these tumors which otherwise have no cure.

## Supplementary Material

vdae058_suppl_Supplementary_Materials

## Data Availability

The data will be made available upon reasonable request. Please reach out to the corresponding author (Y.R.).
