# Supplementary material for: Matrix metalloproteinase 9: An emerging biomarker for classification of adherent vestibular schwannoma
Source: Neurooncol Adv. 2024 Apr 20;6(1):vdae058. doi: 10.1093/noajnl/vdae058 (PMC11181934; doi:10.1093/noajnl/vdae058)
Supplement: vdae058_suppl_Supplementary_Materials [file vdae058_suppl_supplementary_materials.docx]

**Matrix metalloproteinase 9: an emerging biomarker for classification of adherent vestibular schwannoma**

**Authors:** Han TN. Nguyen, Bailey H. Duhon, Hsuan-Chih Kuo, Melanie Fisher, Olivia M. Brickey, Lisa Zhang, Jose J. Otero, Daniel M. Prevedello, Oliver F. Adunka, and Yin Ren

**Supplementary Materials and Methods**

- Supplementary Methods and Materials
- Supplementary Figure Legends
- Supplementary Table Legends
- Supplementary Figures
- Supplementary Tables

**SUPPLEMENTARY METHODS AND MATERIALS**

**Human VS Samples**

Tumors used in the retrospective discovery patient cohort consisted of formalin-fixed, paraffin-embedded tissue from archived tumor bank at Ohio State University (collected between 2010 and 2019). Tumors in the prospective validation cohort consisted of fresh tumor specimens and primary cultures derived from patients with sporadic VS undergoing surgery between June 2022 and July 2023. For histological analysis, a piece of tumor tissue was flash frozen in OCT solution on dry ice and stored in -80°C. In some cases, additional tumor pieces were fixed in 10% formalin and embedded in paraffin. Consecutive histological sections were cut with a thickness of 5 μm, and one section from each tumor was stained with hematoxylin and eosin and examined by an independent neuropathologist to verify that sufficient VS tissues were present.

Details on primary VS culture have been described previously.^1,2^ Briefly, freshly harvested VS specimen was rinsed in PBS, sharply dissected into chunks less than 2 mm in diameter, treated with collagenase and maintained in Dulbecco’s Modified Eagle’s medium (DMEM) with Hams’ F12 mixture, 10% fetal bovine serum (FBS), 1% Penicillin/Streptomycin (Pen/Strep) and 1% Glutamax (Life Technologies, Carlsbad, CA, USA). Cells were passaged when confluency reached over 80%. Collection of tumor conditioned media was performed after 72 hours.

**Clinical data analysis**

Tumor volume was calculated on the most recent preoperative MRI with the modified ellipsoid formula: 4/3×π×½(W×L×H), where W, L, H represent the width, length, and height of the tumor in all three axes. All operative notes were reviewed independently by two investigators (YR and BHD) to ensure congruence. Any discrepancies were discussed amongst the surgical team involved. The extent of tumor resection was defined as follows: in every case, the goal was to achieve gross total resection (GTR) where all visible tumor was removed. The decision to perform either a near-total resection (NTR), where the residual tumor was no greater than 5 x 5 x 2 mm and was typically left along the facial nerve or brainstem to preserve function, or a sub-total resection (STR), where more residual tumor was left in-situ than those after NTR, was made solely intraoperatively based on factors such as the integrity of the facial nerve and the degree of tumor adherence to the brainstem. Facial nerve function was graded using the standardized House-Brackmann scale and obtained at a minimum of 6 months postoperatively. Grade I represented normal facial nerve function and grade VI represented complete facial paralysis.

**Cell culture**

Mouse NF2^-/-^ schwannoma cells (MD-MSC), immortalized human schwannoma cells derived from a patient with NF2 (HEI-193), wild-type mouse Schwann cells (MSC) and human Schwann cells (HSC) were cultured in DMEM supplemented with 10% fetal bovine serum (FBS, Gibco), 1% penicillin/streptomycin, and 10 µM forskolin. All cells were kept at humidified 37^0^C and 5% CO_2_.

**Animal studies**

For *in vivo* studies, athymic nude mice (Jackson Labs, strain code: 007850, homozygous) at ages 6-8 weeks were obtained under the protocol approved by The Ohio State University Institutional Animal Care and Use Committee (2022A00000043). For the tumor allograft model, the sciatic nerve was exposed in the standard fashion and up to 10,000 tumor cells suspended in Matrigel and DMEM was slowly injected into the sciatic nerve sheath using a 27G Hamilton syringe. Tumor growth was monitored on regular intervals for up to 3 weeks, at which point the tumors were harvested and sectioned into equal portions for histology and molecular analysis. Tumors for IHC studies were fixed in 10% formalin overnight, suspended in 30% sucrose, then embedded in OCT. The remaining tissue was placed in RNAlater and stored at –80^0^C.

For evaluation of therapeutic effects of MMP9 inhibition, MD-MSC cells (1×10^6^/mL) were suspended in Matrigel (BD Biosciences) and 100 µL was injected subcutaneously into the flanks of 4-5 week-old female nude mice. Mice were randomized into four treatment groups each comprising 4 to 5 animals. The groups were as follows: control group receiving saline (vehicle), MMP9-IN-I at 3 mg/kg, Silvestrol at 1.5 mg/kg, and Silvestrol at 3mg/kg. Animals were treated on day 3 & 4 after cell implantation by intraperitoneal injection. Mice were weighed and tumor volumes were measured every other day and calculated by the ellipsoid formula [4/3 pi × L/2 × W/2 × H/2].

**nCounter gene expression analysis**

Transcriptomic analysis of murine schwannoma allografts was conducted using the nCounter analysis system (NanoString Technologies, Seattle, WA, USA). This system quantifies the relative abundance of mRNA transcript of interest through a multiplexed hybridization assay and digital readouts of fluorescent barcoded probes hybridized to transcripts. A custom nCounter CodeSet (NanoString Technologies) was employed, comprising a biotinylated capture probe for 100 target genes involved in extracellular matrix remodeling, tissue hypoxia, inflammation, cytokine and chemokine signaling, immune cell infiltration and cell-cell adhesion, and four reference housekeeping genes (**Supplementary Table 3**).

The hybridized samples were loaded into the nCounter Prep Station for post-hybridization processing, purification and immobilization. Target mRNA quantification within each sample was then performed using nCounterTM Digital Analyzer. Quantitative expression data were analyzed using nSolver Analysis Software v2.0. Gene expression data was filtered based on quality control (QC) criteria per manufacturer's guidelines. Subsequently, sample counts that met QC criteria were subjected to normalization, employing a set of four reference housekeeping genes (*ABCF1, GAPDH, HPRT* and *TUBB*). The genes measured below the average negative cutoff level plus 1.5-fold the standard deviation of the negative background were excluded resulting in the removal of nine genes and the retention of 91 genes. We determined the percentage of genes falling below the negative background level, revealing that none of the samples exhibited more than 40% of genes below the background threshold. All genes were normalized using the geometric mean and then subjected to a log2 transformation to obtain the final expression levels.

Student’s t-test was used to compare normalized expression values between groups. Unsupervised hierarchical clustering was performed to analyze of gene expression for the differentially expressed genes using the Euclidean distance, the heatmap shows the median-normalized expression of individual genes across all samples. Principal component analysis was performed. A list candidate list of differential expressed gene sets was identified using a volcano plot that showed > 4-fold or <-4-fold differential expression. For pathway enrichment analysis, we used the DAVID Bioinformatics Resources tool to perform KEGG pathway analysis with a p-value cutoff of 0.05. Gene Ontology Biologic Process Enrichment analysis was performed using the ‘compareCluster’ function from ClusterProfiler (v3.18.1), with the top 25 DEGs ranked by average Log2 FC. For proteases, we filtered the gene list using the MEROPS database (<https://www.ebi.ac.uk/merops/>), a published compendium of all known endopeptidases and inhibitors within the human genome.

**Analysis of microarray and scRNA-seq data**

Three published microarray datasets (GSE141801, GSE108524 and GSE39645) were used. Specifically, GSE141801 contained 36 sporadic tumors and 7 controls of vestibular nerve tissue, GSE108524 dataset containing 10 sporadic VS and 4 control nerves, and GSE39645 contained 28 sporadic VS and 8 control nerves. Expression data was extracted using Geo2R and log2 normalized. Labeling of each sample was obtained from published data and separated into schwannoma vs. normal nerves. In addition, DEGs from scRNA-seq of nonmyelin-forming Schwann cell cluster (nmSC) and myelin-forming Schwann cell cluster (myeSC) were obtained. A linear model was fitted to the data using limma’s (v3.46.0) ‘arrayWeights’ and ‘lmFit’ functions respectively. Empirical Bayes statistics were used for differential expression analysis between normal and tumor samples. The top 30-50 differentially expressed protease genes between schwannomas and normal nerves were identified by cross-referencing DEGs with MEROPS and further visualized using heatmaps GraphPad Prism (v.10.3, Boston, MA).

**Immunoblotting**

Cells were lysed in protein extraction buffer (50 mM HEPES, 1% Triton-X-100, 0.5% sodium deoxycholate, 0.1% SDS, 0.5 mM EDTA, 50 mM NaF, and protease cocktail inhibitor (Cell signaling). Proteins were separated by either 4–12% acrylamide Bis-Tris and were transferred onto a PVDF membrane (Immobilon-P, Millipore), blocked with Intercept (TBS) blocking buffer (Li-COR), incubated with primary antibodies MMP9 (1:1000, Abcam ab13786); eIF4A (1:1000, Cell Signaling Technologies, C32B4); eIF4E (1:1000, Cell Signaling Technologies, 9742); GAPDH (1:1000, Cell Signaling Technologies 97166S ) overnight at 4 °C, and probed with 800nm and 680nm secondary antibodies (1:5000, Li-COR, IRDye®800CW); (1:5000, Li-COR IRDye®680RD). Signal was visualized using the Li-COR Odyssey FC system.

**Immunofluorescence (IF)** **and** **Immunohistochemical (IHC) staining**

FFPE sections were deparaffinized in xylene and rehydrated using a series of graded ethanol washes. The tissue underwent antigen retrieval with Tris-EDTA buffer (pH 9), quenched with hydrogen peroxide and blocked with 5% goat or donkey serum. The following primary antibodies were used: MMP9 (1:100, ThermoFisher MA532705); SOX10 (1:100, Novus NBP2-44475); CD31 (1:200, Cell Signaling Technologies 3518); eIF4E (1:200, Abcam ab33768). For mouse tissue and cells, the mouse-specific MMP9 antibody (1: 100, R&D systems AF909) and SOX-10 (1:250, Abcam Ab155279) were used. After incubation overnight at 4°C, the slide was washed in PBS followed with an appropriate secondary antibody. For IHC, the slides were visualized using the 3,3’-diaminobenzidine (DAB) HRP substrate kit (Vector Labs), washed in H_2_O, and counterstained with hematoxylin.

We obtained whole digital slides using a Zeiss AxioScan.Z1 slide scanner (bright field, 40x magnification, 0.25 um/pixel resolution) and viewed them with Aperio ImageScope v.12 software (Leica Biosystems). For MMP-9 analysis, the whole slide was captured as 16-bit tiled TIFF images. Color deconvolution was applied to each image to isolate the region of interest, while excluding non-tumor areas and artifacts. We proceeded to compute and standardize the area under the curve (AUC) based on pixel intensity histograms for the regions of interest (ROIs). This process was iterated across all tiles, and the resultant average count of positive pixels was normalized relative to the overall tumor area.

To quantify perivascular MMP9 staining, representative ROIs were selected from 40x high-power fields from digital slide images in a blinded fashion. The MMP-9 perivascular intensity was defined as the intensity of MMP-9 staining around the blood vessel, whereas the parenchymal MMP-9 intensity was defined as the intensity of MMP-9 staining in areas away from blood vessels within the tumor parenchyma. For each tumor, approximately 12-15 representative ROIs were chosen, and the intensities were averaged. To quantify the perivascular expression of MMP9, we conducted immunohistochemical analysis on serial tumor sections, categorizing tumors into adherent and non-adherent groups (n = 5 each). Consecutive sections were for MMP9 and CD31/PECAM-1. Regions of interest were identified from CD31+ areas from randomly selected high-powered fields. For each CD31+ vessel, MMP9 expression was evaluated as either above (MMP9+) or below (MMP9-) threshold. We then compared the MMP9 co-localization with CD31+ endothelium between the two groups.

After IF staining, slides were imaged with a Olympus FV3000 Spectral Confocal Laser Scanning Microscope (Olympus). Images at 40-60x were merged into z-stacks and the fluorescent signal intensity for MMP-9 was calculated after subtracting background. Fiji imageJ (NIH) was used to measure the mean fluorescence intensity in the region of interest. The multi-colored image was separated into individual channels and both areas of interest and background were manually selected. At least 10-15 representative ROIs were taken from each slide. All image analyses were performed blinded to the experimental condition.

**Flow cytometry**

HEI-193 cells were treated with TNF-α (40 ng/mL) for 24 hours. Subsequently, 1×10^6^ cells were suspended in FACS buffer (PBS, 0.5% BSA, 0.1% NaN3) and blocked with Fc Blocking buffer for 30 minutes on ice. After three PBS washes, cells were incubated with mouse anti-N-Cadherin antibody (5µl/10^6^ cells, Abcam ab93525) or mouse αvβ3 antibody (2.5µg/10^6^ cells, Novus 23C6) for 1 hour on ice. Surface protein expression was compared to unstained cells or cells not treated with TNF-α and analyzed on FlowJo (Becton, Dickinson & Company).

**Enzyme-linked immunosorbent assay (ELISA)**

  The amount of tumor secreted MMP-9 in conditioned media of primary cultures was quantified using a human MMP-9 ELISA kit (Abcam Ab246539). Similarly, the level of MMP9 secreted by mouse schwannoma cells was determined using a mouse MMP9 ELISA kit (Abcam ab253227) according to the manufacturer’s instructions. Every sample was run in triplicate.

**Quantitative RT-PCR**

RNA was extracted using the RNeasy Mini kit (Qiagen). Briefly, 1µg of RNA was used to synthesized cDNA using iScript™ cDNA Synthesis Kit (Bio-Rad). cDNA was amplified by qPCR in SYBR Green qPCR Master Mix (Bio-Rad) using a Bio-Rad CFX96 system. Analysis of expression was then performed using the web based RT2 profiler PCR Array data analysis software (Qiagen). Triplicate reactions for the gene of interest and the endogenous control were performed separately on the same cDNA samples. For data analysis, the melting curves were verified and only curves with one melting peak were used. CT values were geometrically averaged and used for ΔΔC_T_ calculations. The relative change in gene expression was calculated as 2^−Δ(ΔCT^). The list of primers is shown in **Supplementary Table 9**.

**Multiplex proteomic screening assay (SOMAScan)**

The SOMAscan assay employed 1,305 biotinylated aptamers (SOMAmers) targeting 47% secreted proteins, 28% extracellular domains, and 25% intracellular proteins to measure protein levels in complex biological specimens. These proteins span a range of biological categories, such as receptors, kinases, cytokines, growth factors, hormones, and structural proteins. The SOMAscan used samples from 6 patients with VS – 3 had non-adherent tumors that separated from the brainstem with ease and 3 with densely adherent tumors that lost arachnoid separations between the tumor and brainstem. Six healthy donors who were age- and gender-matched to VS patients were included as controls. Mean and median fold changes of protein expression was calculated for proteins with statistically significant differences between tumor patients and healthy donors, as well as between adherent tumor patients and healthy donors. *P* value for protein expression between groups and control was < 0.05 to be considered significant dysregulated. The heatmap was generated the most significant dysregulated proteases using GraphPad Prism**.** ROC analysis was used to determine the best discriminatory plasma biomarker.

**Transwell cellular migration and invasion assay**

A trans-well system (5-μm pore size, Corning) was engineered to assess the migratory ability of human and mouse schwannoma cells *in vitro*. Briefly, rat-tail collagen I was used to coat the top well (1 mg/mL for 1 hour in 37 °C). A total of 1×10^5^ human schwannoma cells was plated on the upper insert in DMEM without supplement. The lower compartment was filled with DMEM with 10% FBS and 1% penicillin/streptomycin. After culturing cells for 24 hours, cells were stimulated with recombinant MMP-9, recombinant TNF-α at 5, 20 or 40 ng/mL, small molecule protease inhibitors (SB-3CT and MMP9-IN-I), or silvestrol. The cells were allowed to invade through collagen matrix for 24 hours at 37 °C. The cells were collected with a cotton swab, fixed with 4% paraformaldehyde, and permeabilized. The cell was considered to have invaded if they were present in the membrane after DAPI staining. Similar experiments were performed with MD-MSC cells. At least 10-15 representative ROIs were taken. All image analyses were performed in a single blinded manner. The images were thresholded and the watershed algorithm was applied in succession to segregate proximate objects. The cell-stained DAPI was characterized using the "Analyze Particles" function (ImageJ).

**MTT Assay**

HEI-193 cells were plated in a 96-well plate at 5×10^3^. Cells were then treated with TNF-α (10, 20, 40, 100) ng/mL for 12 hours. The MTT assay was conducted according to the manufacturer’s protocol (Abcam Ab211091). Briefly, 50 µL of serum-free media and 50 µL of MTT solution were added, followed by a 3-hour incubation at 37°C. Afterwards, 150 µL of DMSO was added and OD590 nm was measured.

**Statistical Analysis**

In our in vitro studies, randomization was not feasible in the experimental design. Blinding of investigators during both experimentation and outcome assessment was not implemented for *in vitro* studies given the nature of our experimental setup. Continuous factors were summarized with means and ranges, categorical features were summarized with frequency counts and percentages. Unless otherwise noted, non-parametric one-way ANOVA with Dunnett's multiple comparisons test and non-parametric two-way ANOVA with Tukey's multiple comparisons test were employed. Pearson's correlation test was used for quantifying correlations. Summary data in figures are presented as mean ± standard deviation. The Benjamini– Hochberg correction for multiple comparisons was performed. Two-tailed independent Student’s t-test or Mann-Whitney U test (two-tailed) was used for binary comparisons. ROC analysis was utilized to evaluate the diagnostic potential of significantly elevated candidate plasma biomarkers. ROC of biomarker combinations were calculated using binary logistic regression. Statistical analyses were performed using SPSS (IBM, Armonk, NY, USA) and analysis of immunohistochemical staining was performed with GraphPad Prism 9.5.0 (San Diego, CA, USA). P-values < 0.05 were considered statistically significant.

**REFERENCES**

**1.** Wu L, Vasilijic S, Sun Y, et al. Losartan prevents tumor-induced hearing loss and augments radiation efficacy in NF2 schwannoma rodent models. *Science translational medicine.* 2021; 13(602):eabd4816.

**2.** Landegger LD, Sagers JE, Dilwali S, Fujita T, Sahin MI, Stankovic KM. A unified methodological framework for vestibular schwannoma research. *JoVE (Journal of Visualized Experiments).* 2017(124):e55827.

**3.** Gugel I, Ebner FH, Grimm F, et al. Contribution of mTOR and PTEN to radioresistance in sporadic and NF2-associated vestibular schwannomas: a microarray and pathway analysis. *Cancers.* 2020; 12(1):177.

**4.** Torres-Martin M, Lassaletta L, San-Roman-Montero J, et al. Microarray analysis of gene expression in vestibular schwannomas reveals SPP1/MET signaling pathway and androgen receptor deregulation. *International journal of oncology.* 2013; 42(3):848-862.

**5.** Zhao Y, Liu P, Zhang N, et al. Targeting the cMET pathway augments radiation response without adverse effect on hearing in NF2 schwannoma models. *Proceedings of the National Academy of Sciences.* 2018; 115(9):E2077-E2084.

**SUPPLEMENTARY FIGURE LEGENDS**

**Supplementary Figure 1. Transcriptomic analysis of human VS.** (A) Heatmap comparing mRNA expression of top 50 differentially expressed proteases between VS (n=28) and normal nerves (n=8) from published microarray data (Torres-Martin *et al*., GSE39645). (B) Heatmap comparing expression of top 38 differentially expressed proteases between VS (n=10) and normal nerves (n=4) from published microarray data (Zhao *et al*., GSE108524). (C) Two-dimensional unsupervised hierarchical clustering of log2 transformed z-scored data of implanted tumors and WT-MSCs cells. (D) Dot plot showing GO enrichment analysis for top DEGs from murine schwannoma allograft analysis. (E) Dot plot showing KEGG pathway enrichment analysis for top DEGs in murine schwannomas.

**Supplementary Figure 2.** **Expression of** ***TIMP1-4* in murine schwannoma allografts and spatial localization of MMP-9 in human VS tissue.** (A) Relative *MMP9* and *TIMP1* through *TIMP4* gene expression in schwannoma allografts by qRT-PCR. *TIMP1* expression was elevated by over 30-fold and *TIMP-2* by over 10-fold, whereas *TIMP3* was reduced by 2-fold and *TIMP4* by 200-fold. Data from 3 independent experiments are shown as mean ± SD. NS, not significant **p*<0.05, ***p*<0.002 ****p*<0.0002, *****p*<0.0001 by one way ANOVA with post-hoc Tukey test. (B) Representative immunofluorescence images of human VS tissue showing MMP-9 (green) and schwannoma cells (SOX-10, purple). Scale bar, 100 µm.

**Supplementary Figure 3**. **Some large, non-adherent human VSs express low levels of MMP9.** Representative IHC staining of MMP9 in 3 large, non-adherent vestibular schwannomas from patients. Scale bar, 100 µm.

**Supplementary Figure 4. MMP9 is a Tumor Classifying Biomarker for Adherent VS**. (A) Consort flow diagram of the prospective validation patient cohort. (B) ROC analysis comparing clinical and radiographic variables to classify adherent from non-adherent VS. 4-marker, brainstem compression + CSF cleft + cystic change + MMP9 level; 5-marker, brainstem compression + CSF cleft + cystic change + MMP9 level + tumor volume. (B) ROC analysis of patients with medium-to-large sized tumors (maximum diameter > 15 mm). 3-marker, brainstem compression + CSF cleft + cystic change; 4-marker, brainstem compression + CSF cleft + cystic change + MMP9 level.

**Supplementary Figure 5. MMP9 expression is regulated by components of the eIF4 complex and MMP9 enhances cell adhesion *in vitro*.** (A) eIF4E protein expression is inhibited by silvestrol. (B) Quantification of eIF4E band intensities from immunoblot in A. (C) Representative flow cytometry histograms showing increased expression of αvβ3 with TNF-α treatment in HEI-193 cells *in vitro*. (D) Flow cytometry analysis showing enhanced N-cadherin expression following with TNF-α treatment in HEI-193 cells *in vitro*.  **(**E**)** MTT assay showing HEI-193 viability expressed as the percentage of optical density. TNF-α treatment slightly decreased cell viability in a dose-dependent manner. Data from 3 independent experiments are shown as mean ± SD.  **p*<0.05, ***p*<0.002, ns not significant, by one way ANOVA with post-hoc Tukey test.

**Supplementary Figure 6. MMP9 enhances human and mouse NF2^-/-^schwannoma cell invasion *in vitro*.** (A) Representative immunofluorescence images from transwell assays. Membranes were stained with DAPI. No effect on HSC motility was observed with TNF-α or protease inhibitors (top row). TNF-α (10 to 40 ng/mL) enhanced HEI-193 cell invasion through collagen in a dose-dependent manner, whereas small molecule inhibitors of protease activity (SB-3CT, MMP9-IN-I) abrogated invasion (bottom row). Six biologically independent experiments were performed. Scale bar, 100 µm. (B) A similar invasion assay was performed with WT-MSC (top) and MD-MSC cells (bottom). Scale bar, 100 µm. (C) Quantification of the number of invaded cells from (B). TNF-α significantly enhanced the number of invading MD-MSC cells by 7-fold but did not affect WT-MSCs. Data from 3 independent experiments are shown as mean ± SD.*****p*<0.0001 TNF-α 40ng/mL vs. untreated; ####*p*<0.0001 TNF-α 40ng/mL vs SB-3CT and MMP9-IN-I respectively, one way ANOVA with post-hoc Tukey test. (D) Transwell assay to determine the effect of MMP9 on murine schwannoma cell invasion. Scale bar, 100 µm. (E) Quantification of (D), exogenous MMP9 treatment significantly increased the number of invading MD-MSC cells whereas no effect was observed in WT-MSCs. Mean ± SD of N=3 biologically independent samples are shown, ***p*<0.002, *****p*<0.0001, by two-way ANOVA with Bonferroni-Dunn post-hoc analysis. (F) Representative immunofluorescence images from transwell assays show that treatment with TNF-α (40ng/mL) increased cellular invasion by 10-fold, and subsequent administration of Silvestrol or MMP9-IN-I significantly reduced cellular invasion. (G) Quantification of invasion studies from F. Mean ± SD of N=3 biologically independent samples are shown, *****p*<0.0001, by one-way ANOVA with Bonferroni-Dunn post-hoc analysis.

**Supplementary Figure 7.** **MMP9-IN-I treatment** **did not affect animal body weight.** Effects of MMP9-IN-I and silvestrol treatment on body weight of mice bearing subcutaneous schwannoma allografts. (Saline control, Blue; MMP9-IN-I 3 mg/kg, red; silvestrol 1,5mg/kg, green and silvestrol 3mg/kg, purple). Ns, not significant (MMP9-IN-I vs. Saline control), ***p<*0.0031(silvestrol 1.5 mg/kg vs. Saline control) and ****p*<0.0006 (silvestrol 3 mg/kg vs. Saline control).

**SUPPLEMENTARY TABLE LEGENDS**

**Supplementary Table 1.** List of top proteases enriched in myelinating Schwann cell and non-myelinating Schwann cell clusters from the scRNA-seq dataset.

**Supplementary Table 2.** Expression of proteases from curated microarray datasets. The first shows the ranking of the top 25 upregulated and 22 downregulated expressed proteases between VS (n=36) and normal nerve (n=7) from Gugel *et al* (GSE141801).^3^ The second shows the top 30 differentially expressed proteases between VS (n=28) and normal nerves (n=8) from Torres-Martin *et al* (GSE39645)^4^ and the third list is between VS (n=10) and normal nerves (n=4) from published microarray data (Zhao *et al*., GSE108524).^5^

**Supplementary Table 3.** Transcriptomic analysis of a panel of 100 target genes involved in extracellular matrix remodeling, tissue hypoxia, inflammation, cytokine and chemokine signaling, immune cell infiltration and cell-cell adhesion, and four reference housekeeping genes in schwannoma allografts (MD-MSC tumor), schwannoma cells (MD-MSC) and wild-type Schwann cells (WT-MSC). Log2 fold change values are shown.

**Supplementary Table 4.** List of genes validated from transcriptomic analysis of DEGs comparing implanted tumor versus WT-MSC cells or comparing MD-MSC cells versus WT-MSC cells.

**Supplementary Table 5.** Demographics of patients used in the subanalysis of size-matched adherent and non-adherent VS from the prospective cohort.

**Supplementary Table 6.** Demographics of patients and healthy volunteers whose plasma samples were used for SOMAScan.

**Supplementary Table 7.** Proteomic analysis profiling the top 20 most abundant circulating proteases in plasma of VS patients compared to heathy donors. The second table shows the top 20 most abundant endopeptidases in sub-analysis in patients with adherent VS compared to healthy donors. M, metalloproteases; S, serine proteases; C, cathepsins. P-values are adjusted for multiple comparisons.

**Supplementary Table 8.** Results from the ROC analysis of biomarkers to classify adherent VS including AUC, sensitivity, specificity, negative predictive value and positive predictive value.

**Supplementary Table 9.** Sequence of primers used in qRT-PCR.

**Supplementary Figure 1**

**
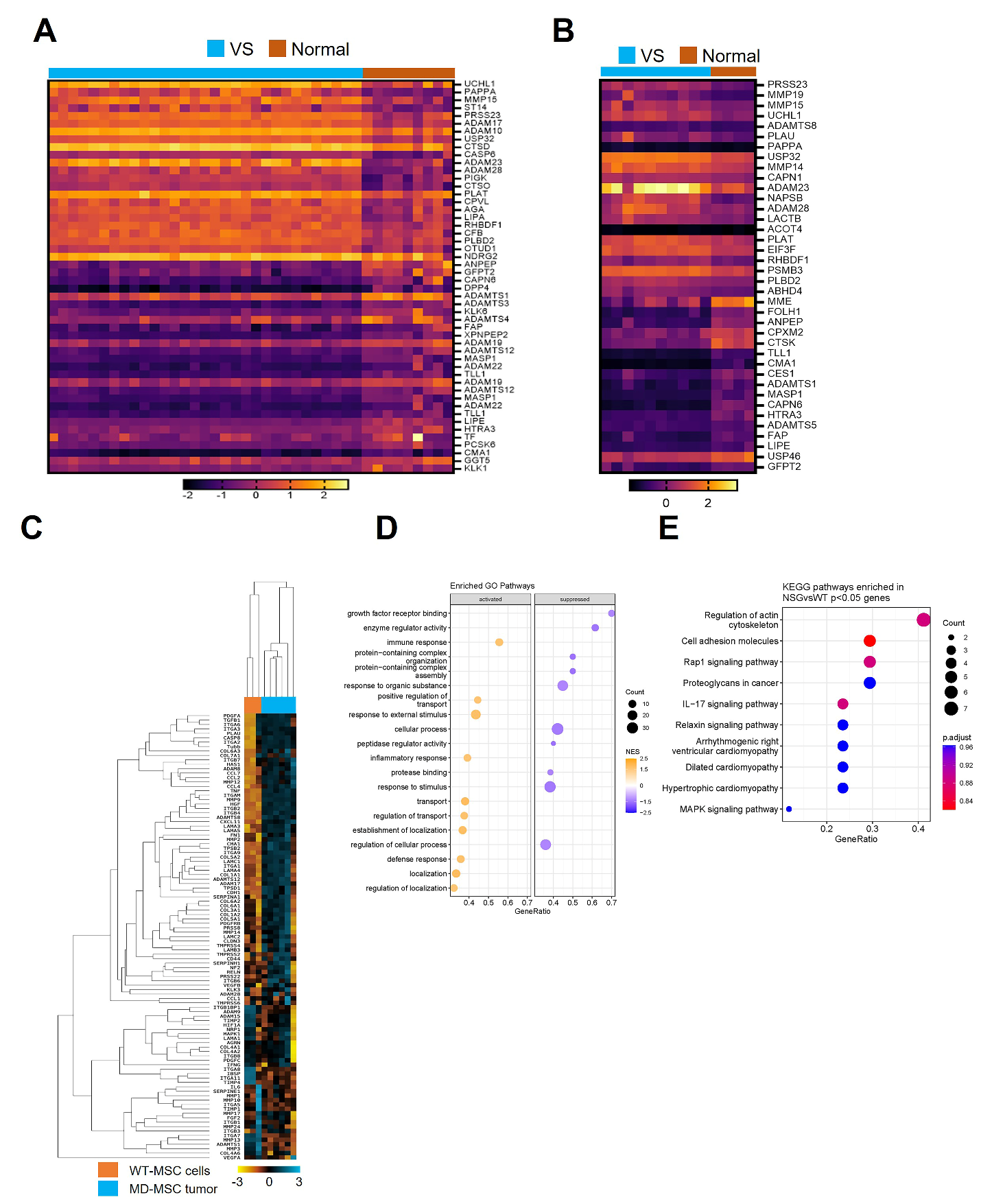
**

**Supplementary Figure 2**

**
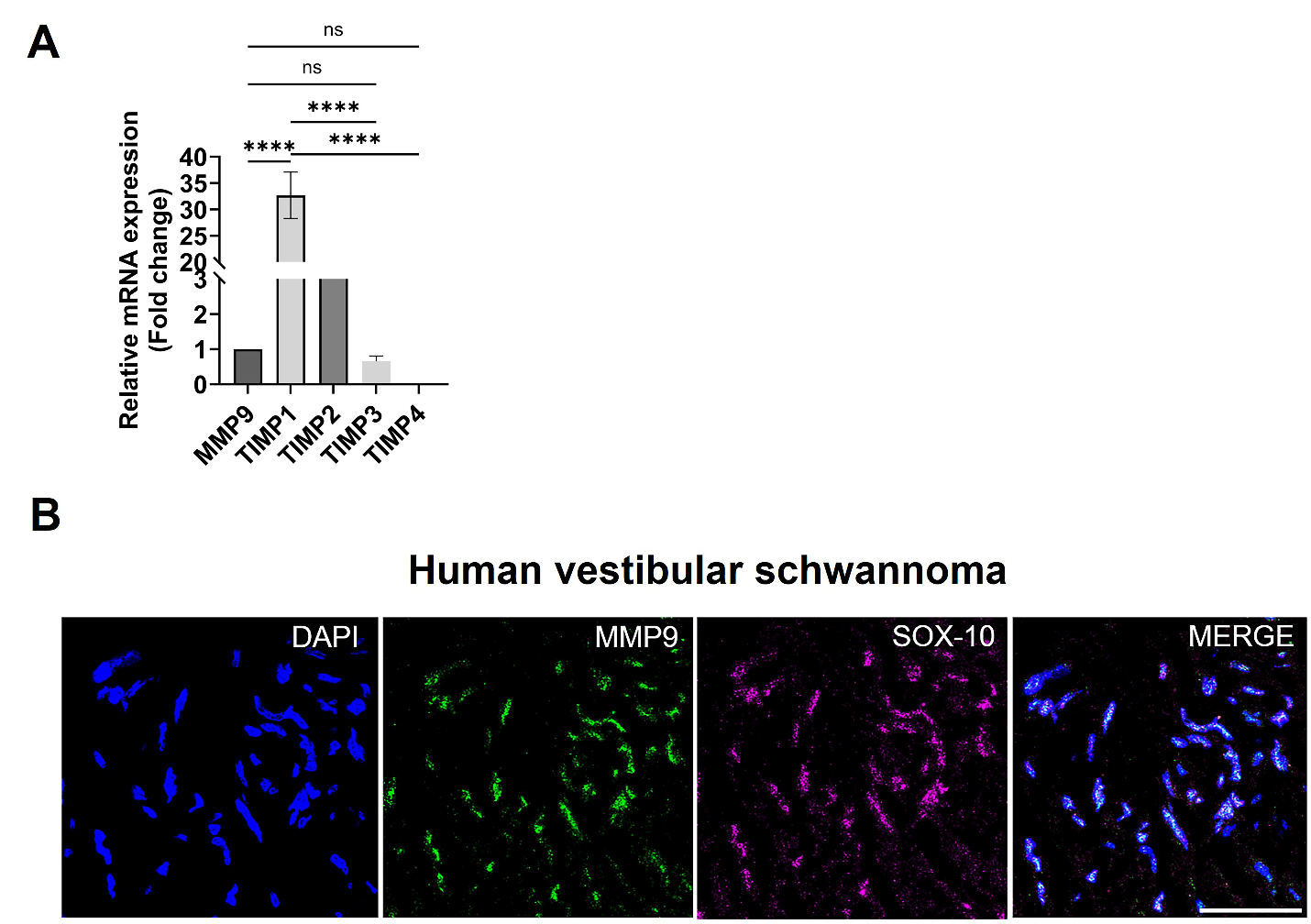
**

**Supplementary Figure 3
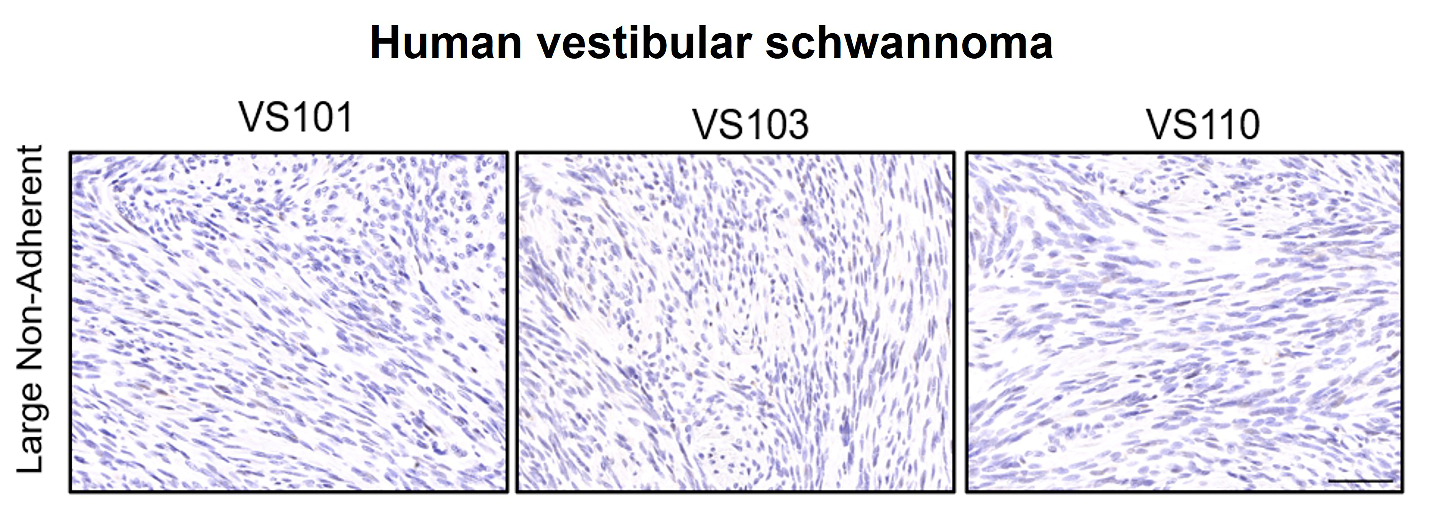
**

**Supplementary Figure 4**

**
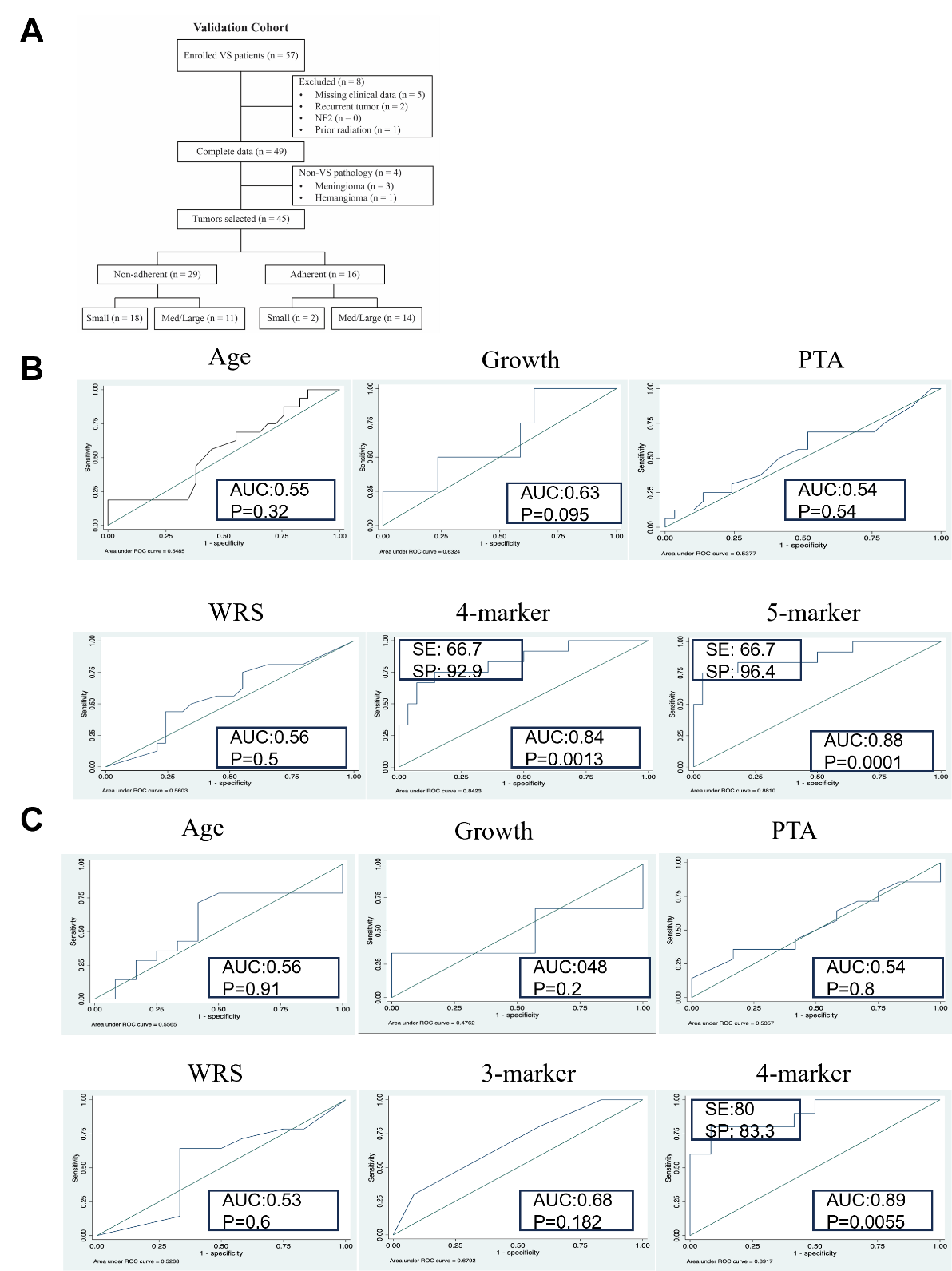
**

**Supplementary Figure 5**

**
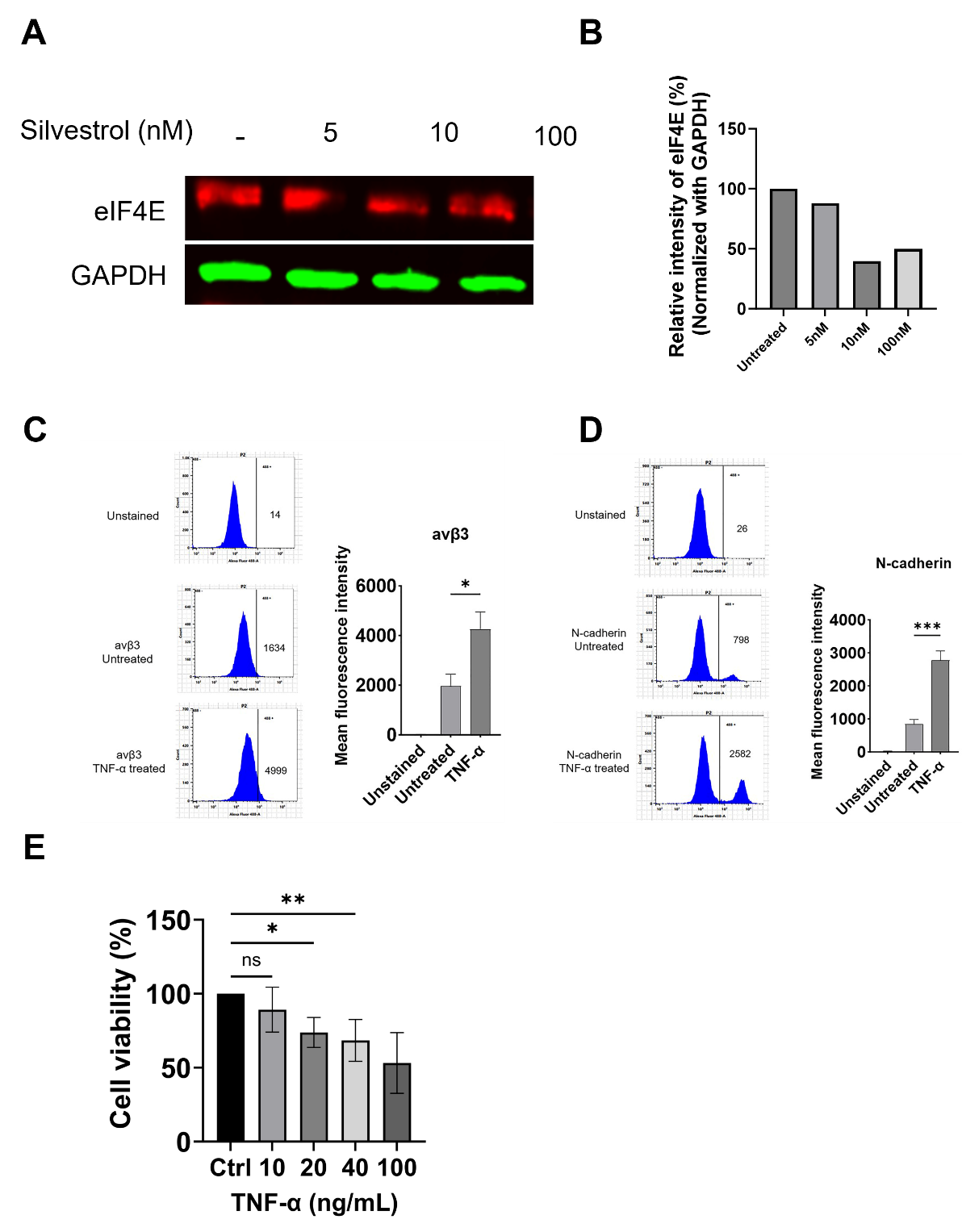
**

**Supplementary Figure 6**

**
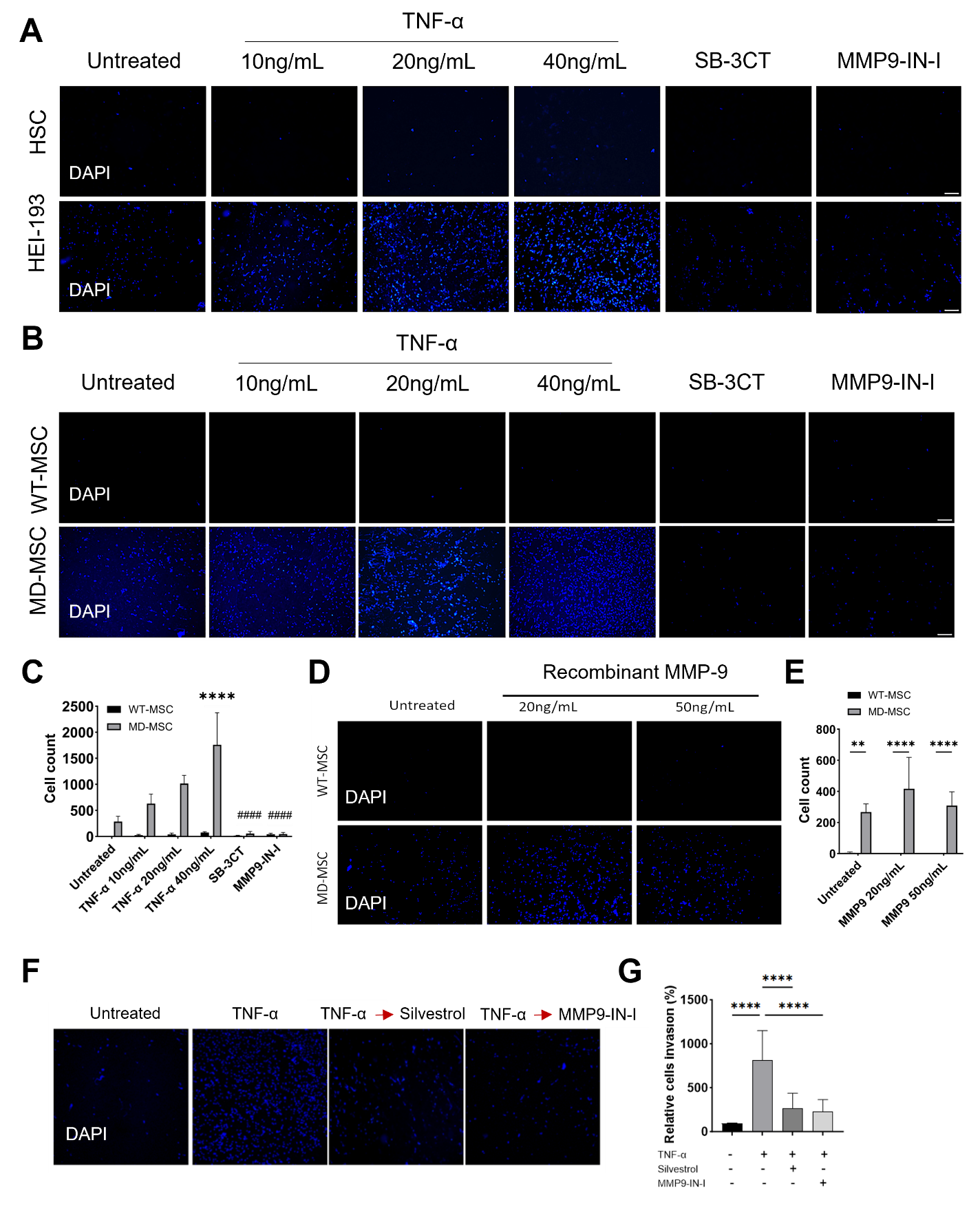
**

**Supplementary Figure 7**

**
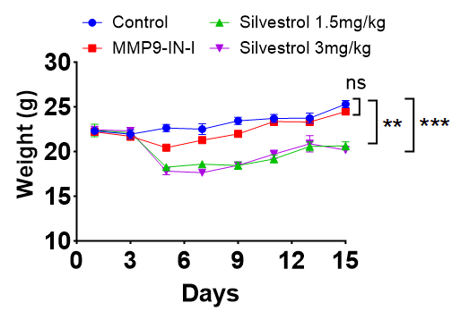
**

**Supplementary Table 1**

| **Rank** | **Gene** | **Cluster** | **p_val** | **Log2FC** |
| --- | --- | --- | --- | --- |
| 1 | ADAMTS9 | myeSC-like Cells | 0 | 2.812 |
| 2 | OTUD7B | myeSC-like Cells | 0 | 2.196 |
| 3 | MME | myeSC-like Cells | 0 | 2.095 |
| 4 | NDRG1 | myeSC-like Cells | 0 | 2.039 |
| 5 | MGLL | myeSC-like Cells | 0 | 1.988 |
| 6 | NAALADL2 | myeSC-like Cells | 0 | 1.930 |
| 7 | BACE2 | myeSC-like Cells | 0 | 1.695 |
| 8 | DHH | myeSC-like Cells | 0 | 1.350 |
| 9 | TMPRSS5 | myeSC-like Cells | 0 | 1.331 |
| 10 | SCRN1 | myeSC-like Cells | 0 | 1.204 |
| 11 | CPXM2 | myeSC-like Cells | 0 | 1.154 |
| 12 | PLAT | myeSC-like Cells | 0 | 1.000 |
|  |  |  |  |  |
| **Rank** | **Gene** | **Cluster** | **p_val** | **Log2FC** |
| 1 | ADAM23 | nmSC-like Cells | 0 | 3.718 |
| 2 | IMMP2L | nmSC-like Cells | 0 | 2.701 |
| 3 | NLGN4X | nmSC-like Cells | 0 | 2.343 |
| 4 | NAALADL2 | nmSC-like Cells | 0 | 2.165 |
| 5 | NLGN1 | nmSC-like Cells | 0 | 1.987 |
| 6 | OTUD7B | nmSC-like Cells | 0 | 1.984 |
| 7 | PAPPA | nmSC-like Cells | 0 | 1.961 |
| 8 | PRSS23 | nmSC-like Cells | 0 | 1.673 |
| 9 | ADAMTS9 | nmSC-like Cells | 0 | 1.518 |
| 10 | NDRG1 | nmSC-like Cells | 0 | 1.363 |
| 11 | NDRG2 | nmSC-like Cells | 0 | 1.288 |
| 12 | PLAT | nmSC-like Cells | 0 | 1.231 |
| 13 | ECE1 | nmSC-like Cells | 0 | 1.128 |
| 14 | MMP28 | nmSC-like Cells |  | 1.090 |
| 15 | UCHL1 | nmSC-like Cells | 0 | 1.024 |
| 16 | USP32 | nmSC-like Cells | 0 | 1.006 |

**Supplementary Table 2**

**GSE141801**

| **Rank** | **Gene.Symbol** | **ID** | **adj.P.Val** | **P.Value** | **t** | **B** | **logFC** |
| --- | --- | --- | --- | --- | --- | --- | --- |
| 1 | ADAM23 | 11736770_a_at | 1.32E-15 | 1.65E-18 | 14.683 | 31.972 | 4.424 |
| 2 | ST14 | 11715965_at | 6.05E-07 | 4.02E-08 | 6.637 | 8.340 | 3.339 |
| 3 | CTSS | 11749589_x_at | 9.88E-10 | 2.16E-11 | 8.916 | 15.801 | 2.761 |
| 4 | ADAM28 | 11736747_x_at | 7.25E-08 | 3.37E-09 | 7.374 | 10.792 | 2.533 |
| 5 | MMP19 | 11719531_a_at | 1.00E-10 | 1.44E-12 | 9.775 | 18.486 | 2.492 |
| 6 | PAPPA | 11727014_at | 4.88E-06 | 4.49E-07 | 5.922 | 5.958 | 2.368 |
| 7 | LIPA | 11745775_a_at | 4.99E-08 | 2.17E-09 | 7.506 | 11.227 | 2.167 |
| 8 | CTSZ | 11726594_at | 7.26E-10 | 1.51E-11 | 9.027 | 16.152 | 2.156 |
| 9 | PRSS23 | 11749254_a_at | 1.25E-13 | 4.19E-16 | 12.563 | 26.536 | 2.118 |
| 10 | GZMK | 11728560_at | 1.06E-03 | 2.14E-04 | 4.038 | -0.057 | 1.900 |
| 11 | GZMA | 11720496_at | 2.16E-05 | 2.46E-06 | 5.415 | 4.286 | 1.870 |
| 12 | PLAU | 11717886_a_at | 3.82E-06 | 3.39E-07 | 6.006 | 6.236 | 1.866 |
| 13 | GZMH | 11728125_a_at | 1.73E-04 | 2.66E-05 | 4.695 | 1.962 | 1.823 |
| 14 | ADAMTS8 | 11731704_a_at | 1.08E-03 | 2.19E-04 | 4.031 | -0.080 | 1.794 |
| 15 | MMP14 | 11725989_x_at | 4.62E-12 | 3.51E-14 | 11.005 | 22.163 | 1.762 |
| 16 | ASAH1 | 11740580_at | 1.40E-04 | 2.08E-05 | 4.770 | 2.200 | 1.746 |
| 17 | CARD8 | 11740036_at | 1.55E-11 | 1.54E-13 | 10.508 | 20.701 | 1.641 |
| 18 | AGA | 11723963_a_at | 1.51E-10 | 2.34E-12 | 9.619 | 18.004 | 1.521 |
| 19 | LACTB | 11734305_a_at | 6.07E-09 | 1.81E-10 | 8.259 | 13.693 | 1.513 |
| 20 | NLGN3 | 11726389_a_at | 4.13E-06 | 3.70E-07 | 5.980 | 6.150 | 1.490 |
| 21 | CASP8 | 11728978_x_at | 2.45E-07 | 1.39E-08 | 6.952 | 9.392 | 1.484 |
| 22 | CARD8 | 11747437_a_at | 1.35E-09 | 3.11E-11 | 8.802 | 15.438 | 1.482 |
| 23 | CASP4 | 11748529_x_at | 2.07E-07 | 1.14E-08 | 7.011 | 9.587 | 1.460 |
| 24 | BAI2 | 11721729_a_at | 6.70E-09 | 2.04E-10 | 8.222 | 13.572 | 1.405 |
| 25 | MMP9 | 11719657_a_at | 4.99E-02 | 1.99E-02 | 2.416 | -4.306 | 1.350 |
|  |  |  |  |  |  |  |  |
| **Rank** | **Gene.Symbol** | **ID** | **adj.P.Val** | **P.Value** | **t** | **B** | **logFC** |
| 1 | GFPT2 | 11721134_at | 3.44E-10 | 6.19E-12 | -9.309 | 17.039 | -2.792 |
| 2 | ANPEP | 11717127_a_at | 3.85E-11 | 4.59E-13 | -10.147 | 19.617 | -2.713 |
| 3 | FOLH1 | 11747117_x_at | 7.36E-14 | 2.16E-16 | -12.806 | 27.188 | -2.690 |
| 4 | KLK7 | 11753255_a_at | 1.25E-10 | 1.86E-12 | -9.693 | 18.231 | -2.689 |
| 5 | KLK6 | 11725703_a_at | 1.39E-06 | 1.05E-07 | -6.353 | 7.392 | -2.520 |
| 6 | KLK1 | 11727976_a_at | 2.93E-13 | 1.26E-15 | -12.164 | 25.447 | -2.515 |
| 7 | ADAMTS4 | 11730923_x_at | 2.85E-05 | 3.38E-06 | -5.321 | 3.976 | -2.363 |
| 8 | TF | 11756783_a_at | 5.67E-05 | 7.43E-06 | -5.083 | 3.205 | -2.200 |
| 9 | USP46 | 11725800_a_at | 2.56E-11 | 2.82E-13 | -10.307 | 20.099 | -2.177 |
| 10 | CPXM2 | 11719536_at | 2.50E-03 | 5.80E-04 | -3.711 | -1.013 | -2.159 |
| 11 | CPE | 11743577_s_at | 2.44E-07 | 1.38E-08 | -6.954 | 9.397 | -2.145 |
| 12 | ADAMTS1 | 11754662_s_at | 5.93E-08 | 2.66E-09 | -7.445 | 11.026 | -2.101 |
| 13 | MME | 11741562_a_at | 1.14E-02 | 3.40E-03 | -3.098 | -2.685 | -2.076 |
| 14 | HTRA3 | 11759587_at | 4.20E-09 | 1.17E-10 | -8.393 | 14.126 | -1.922 |
| 15 | GGT5 | 11756254_a_at | 2.53E-06 | 2.11E-07 | -6.147 | 6.705 | -1.716 |
| 16 | DPP4 | 11758194_s_at | 1.72E-05 | 1.91E-06 | -5.492 | 4.537 | -1.697 |
| 17 | NDRG4 | 11736068_x_at | 1.04E-04 | 1.47E-05 | -4.876 | 2.537 | -1.674 |
| 18 | CPXM2 | 11719537_a_at | 3.55E-03 | 8.65E-04 | -3.577 | -1.394 | -1.674 |
| 19 | CES1 | 11721638_s_at | 2.22E-02 | 7.54E-03 | -2.802 | -3.422 | -1.613 |
| 20 | PCSK6 | 11723385_s_at | 7.93E-04 | 1.53E-04 | -4.146 | 0.267 | -1.536 |
| 21 | KLK10 | 11727658_s_at | 1.29E-08 | 4.43E-10 | -7.986 | 12.803 | -1.503 |
| 22 | MUC1 | 11745158_x_at | 1.34E-08 | 4.66E-10 | -7.970 | 12.754 | -1.493 |

**GSE39645**

| **Rank** | **Gene. Symbol** | **ID** | **adj.P.Val** | **P.Value** | **t** | **B** | **logFC** |
| --- | --- | --- | --- | --- | --- | --- | --- |
| 1 | ADAM23 | 8047788 | 2.97E-12 | 1.18E-14 | 11.545 | 23.260 | 3.251 |
| 2 | NLGN4X | 8171205 | 1.55E-15 | 5.29E-19 | 15.459 | 33.045 | 2.547 |
| 3 | UCHL1 | 8094778 | 6.23E-10 | 9.78E-12 | 9.265 | 16.630 | 2.079 |
| 4 | PAPPA | 8157487 | 1.64E-07 | 9.89E-09 | 7.106 | 9.788 | 2.031 |
| 5 | MMP15 | 7996185 | 7.60E-08 | 3.83E-09 | 7.395 | 10.726 | 1.947 |
| 6 | ADAM28 | 8145293 | 1.75E-07 | 1.07E-08 | 7.082 | 9.708 | 1.615 |
| 7 | ST14 | 7945204 | 1.88E-05 | 3.40E-06 | 5.344 | 4.038 | 1.600 |
| 8 | PRSS23 | 7942957 | 5.29E-09 | 1.37E-10 | 8.424 | 14.017 | 1.521 |
| 9 | PIGK | 7917088 | 3.09E-07 | 2.12E-08 | 6.875 | 9.034 | 1.425 |
| 10 | CTSO | 8103389 | 1.26E-08 | 4.20E-10 | 8.074 | 12.911 | 1.404 |
| 11 | ADAM17 | 8050190 | 5.66E-11 | 4.76E-13 | 10.263 | 19.617 | 1.366 |
| 12 | PLAT | 8150509 | 1.24E-08 | 4.09E-10 | 8.083 | 12.937 | 1.357 |
| 13 | CPVL | 8138805 | 2.56E-05 | 5.01E-06 | 5.226 | 3.659 | 1.350 |
| 14 | AGA | 8103834 | 7.70E-08 | 3.91E-09 | 7.389 | 10.705 | 1.347 |
| 15 | LIPA | 7934920 | 7.51E-08 | 3.76E-09 | 7.400 | 10.743 | 1.346 |
| 16 | ADAM10 | 7989224 | 8.49E-10 | 1.41E-11 | 9.147 | 16.267 | 1.319 |
| 17 | NLGN3 | 8168264 | 8.65E-06 | 1.29E-06 | 5.639 | 4.992 | 1.317 |
| 18 | RHBDF1 | 7998136 | 2.37E-08 | 9.15E-10 | 7.834 | 12.141 | 1.281 |
| 19 | USP32 | 8017212 | 2.31E-10 | 2.94E-12 | 9.657 | 17.818 | 1.273 |
| 20 | PREP | 8128572 | 4.76E-09 | 1.21E-10 | 8.463 | 14.143 | 1.248 |
| 21 | USP9X | 8166826 | 4.78E-08 | 2.16E-09 | 7.570 | 11.293 | 1.239 |
| 22 | BRCC3 | 8171006 | 4.70E-07 | 3.49E-08 | 6.725 | 8.543 | 1.219 |
| 23 | CTSD | 7945666 | 2.58E-08 | 1.01E-09 | 7.803 | 12.042 | 1.216 |
| 24 | CFI | 8102328 | 1.52E-03 | 7.53E-04 | 3.633 | -1.190 | 1.197 |
| 25 | CFB | 8118345 | 2.65E-06 | 2.98E-07 | 6.080 | 6.430 | 1.159 |
| 26 | PLBD2 | 7958989 | 1.85E-09 | 3.63E-11 | 8.844 | 15.333 | 1.157 |
| 27 | OTUD1 | 7926677 | 2.56E-06 | 2.86E-07 | 6.092 | 6.469 | 1.141 |
| 28 | NDRG2 | 7977621 | 7.27E-05 | 1.82E-05 | 4.830 | 2.400 | 1.126 |
| 29 | CASP6 | 8102311 | 1.05E-06 | 9.48E-08 | 6.424 | 7.557 | 1.116 |
| 30 | CYLD | 7995552 | 5.56E-08 | 2.62E-09 | 7.511 | 11.103 | 1.098 |
|  |  |  |  |  |  |  |  |
| **Rank** | **Gene. Symbol** | **ID** | **adj.P.Val** | **P.Value** | **t** | **B** | **logFC** |
| 1 | ANPEP | 1.13E-09 | 7991335 | 2.04E-11 | -9.028 | 15.901 | -2.202 |
| 2 | GFPT2 | 4.74E-07 | 8116418 | 3.53E-08 | -6.722 | 8.531 | -1.886 |
| 3 | CPE | 2.15E-05 | 8098204 | 4.03E-06 | -5.293 | 3.871 | -1.824 |
| 4 | CAPN6 | 3.96E-06 | 8174527 | 4.90E-07 | -5.930 | 5.939 | -1.647 |
| 5 | DPP4 | 3.14E-08 | 8056222 | 1.30E-09 | -7.726 | 11.795 | -1.607 |
| 6 | ADAMTS1 | 1.36E-06 | 8069676 | 1.30E-07 | -6.328 | 7.242 | -1.540 |
| 7 | ADAMTS3 | 1.96E-11 | 8100870 | 1.30E-13 | -10.704 | 20.895 | -1.467 |
| 8 | KLK6 | 5.93E-07 | 8038683 | 4.67E-08 | -6.637 | 8.256 | -1.401 |
| 9 | ADAMTS4 | 4.80E-04 | 7921821 | 1.88E-04 | -4.093 | 0.140 | -1.347 |
| 10 | FAP | 3.36E-04 | 8056257 | 1.21E-04 | -4.235 | 0.563 | -1.213 |
| 11 | XPNPEP2 | 5.88E-11 | 8169836 | 5.18E-13 | -10.234 | 19.533 | -1.189 |
| 12 | CFD | 7.47E-05 | 8024062 | 1.88E-05 | -4.820 | 2.367 | -1.188 |
| 13 | ADAM19 | 1.40E-06 | 8115490 | 1.35E-07 | -6.317 | 7.208 | -1.167 |
| 14 | ADAMTS12 | 2.61E-07 | 8111387 | 1.71E-08 | -6.941 | 9.248 | -1.139 |
| 15 | MASP1 | 2.09E-07 | 8092661 | 1.31E-08 | -7.022 | 9.512 | -1.100 |
| 16 | ADAM22 | 1.34E-05 | 8133983 | 2.23E-06 | -5.472 | 4.450 | -1.095 |
| 17 | TLL1 | 6.74E-09 | 8098214 | 1.85E-10 | -8.330 | 13.721 | -1.094 |
| 18 | CTSG | 1.44E-07 | 7978351 | 8.38E-09 | -7.157 | 9.951 | -1.089 |
| 19 | LIPE | 1.94E-10 | 8037186 | 2.32E-12 | -9.735 | 18.053 | -1.023 |
| 20 | HTRA3 | 2.97E-07 | 8094056 | 2.02E-08 | -6.890 | 9.083 | -0.997 |
| 21 | TF | 4.02E-02 | 8082797 | 2.95E-02 | -2.253 | -4.583 | -0.991 |
| 22 | PCSK6 | 3.15E-07 | 7991602 | 2.17E-08 | -6.869 | 9.013 | -0.965 |
| 23 | CMA1 | 3.42E-06 | 7978343 | 4.06E-07 | -5.986 | 6.123 | -0.941 |
| 24 | ADAMTS18 | 8.57E-08 | 8002941 | 4.47E-09 | -7.348 | 10.573 | -0.935 |
| 25 | CNDP1 | 1.29E-03 | 8021741 | 6.22E-04 | -3.698 | -1.008 | -0.882 |
| 26 | ACE2 | 4.20E-07 | 8171449 | 3.06E-08 | -6.764 | 8.671 | -0.867 |
| 27 | DPYSL5 | 8.49E-05 | 8040725 | 2.22E-05 | -4.769 | 2.207 | -0.847 |
| 28 | GGT5 | 4.39E-04 | 8074991 | 1.69E-04 | -4.127 | 0.242 | -0.846 |
| 29 | KLK1 | 2.61E-04 | 8038633 | 8.91E-05 | -4.332 | 0.857 | -0.833 |
| 30 | DPYSL3 | 1.50E-03 | 8114920 | 7.42E-04 | -3.638 | -1.176 | -0.826 |

**GSE108524**

| **Rank** | **Gene Symbol** | **ID** | **adj.P.Val** | **P.Value** | **t** | **B** | **logFC** |
| --- | --- | --- | --- | --- | --- | --- | --- |
| 1 | ADAM23 | TC02001218.hg.1 | 4.976E-03 | 6.630E-04 | -3.763 | -0.900 | -0.492 |
| 2 | NAPSB | TC19002549.hg.1 | 1.929E-06 | 2.177E-08 | -7.124 | 8.496 | -0.429 |
| 3 | PRSS23 | TC11002811.hg.1 | 3.635E-08 | 1.572E-10 | -8.886 | 13.156 | -0.342 |
| 4 | MMP19 | TC12001587.hg.1 | 1.153E-03 | 9.230E-05 | -4.474 | 1.032 | -0.340 |
| 5 | NLGN3 | TC0X000383.hg.1 | 2.842E-04 | 1.444E-05 | -5.116 | 2.841 | -0.324 |
| 6 | OVCH2 | TC11001373.hg.1 | 3.879E-02 | 1.078E-02 | -2.709 | -3.497 | -0.321 |
| 7 | ADAM28 | TC08000190.hg.1 | 3.143E-03 | 3.538E-04 | -4.000 | -0.268 | -0.263 |
| 8 | MMP15 | TC16000500.hg.1 | 3.954E-03 | 4.838E-04 | -3.888 | -0.569 | -0.261 |
| 9 | UCHL1 | TC04000253.hg.1 | 1.052E-03 | 8.108E-05 | -4.519 | 1.158 | -0.253 |
| 10 | LACTB | TC15000580.hg.1 | 4.821E-06 | 7.151E-08 | -6.964 | 8.059 | -0.247 |
| 11 | ADAMTS8 | TC11002452.hg.1 | 3.702E-03 | 4.435E-04 | -3.919 | -0.485 | -0.246 |
| 12 | PLAU | TC10000475.hg.1 | 4.475E-03 | 5.754E-04 | -3.825 | -0.735 | -0.241 |
| 13 | PREP | TC06001980.hg.1 | 2.457E-05 | 5.965E-07 | -6.218 | 5.970 | -0.227 |
| 14 | NLGN4Y | TC0Y000263.hg.1 | 1.515E-03 | 1.332E-04 | -3.641 | -1.149 | -0.227 |
| 15 | NAPSA | TC19001746.hg.1 | 1.418E-05 | 2.892E-07 | -6.471 | 6.682 | -0.224 |
| 16 | PAPPA | TC09002212.hg.1 | 3.095E-05 | 8.066E-07 | -4.125 | 0.072 | -0.221 |
| 17 | USP32 | TC17001761.hg.1 | 4.099E-07 | 3.019E-09 | -8.117 | 11.177 | -0.206 |
| 18 | MMP14 | TC14000133.hg.1 | 1.312E-05 | 2.598E-07 | -6.508 | 6.788 | -0.206 |
| 19 | ACOT4 | TC14002081.hg.1 | 5.550E-05 | 1.745E-06 | -4.603 | 1.487 | -0.198 |
| 20 | RHBDF2 | TC17001899.hg.1 | 3.628E-04 | 1.987E-05 | -5.006 | 2.529 | -0.186 |
| 21 | PLAT | TC08001175.hg.1 | 9.978E-03 | 1.693E-03 | -3.388 | -1.871 | -0.186 |
| 22 | CAPN1 | TC11000620.hg.1 | 3.067E-07 | 2.173E-09 | -8.058 | 11.018 | -0.182 |
| 23 | USP9X | TC0X000195.hg.1 | 2.229E-07 | 1.419E-09 | -8.401 | 11.920 | -0.179 |
| 24 | PGPEP1 | TC19000355.hg.1 | 5.294E-06 | 8.108E-08 | -6.920 | 7.935 | -0.178 |
| 25 | EIF3F | TC11000154.hg.1 | 8.406E-05 | 3.015E-06 | -5.656 | 4.377 | -0.176 |
| 26 | RHBDF1 | TC16000721.hg.1 | 4.588E-03 | 5.940E-04 | -3.814 | -0.766 | -0.173 |
| 27 | PSMB3 | TC17000466.hg.1 | 8.058E-05 | 2.845E-06 | -5.676 | 4.434 | -0.170 |
| 28 | PLBD2 | TC12000891.hg.1 | 4.304E-05 | 1.255E-06 | -5.959 | 5.238 | -0.169 |
| 29 | ABHD4 | TC14000130.hg.1 | 1.367E-03 | 1.162E-04 | -4.393 | 0.809 | -0.168 |
| 30 | PSMB9 | TC6_ssto_hap7000193.hg.1 | 3.927E-05 | 1.105E-06 | -5.812 | 4.818 | -0.166 |
|  |  |  |  |  |  |  |  |
| **Rank** | **Gene. Symbol** | **ID** | **adj.P.Val** | **P.Value** | **t** | **B** | **logFC** |
| 1 | CFD | TC19000020.hg.1 | 2.960E-12 | 1.931E-15 | 14.360 | 25.026 | 0.750 |
| 2 | MME | TC03000846.hg.1 | 4.751E-04 | 2.843E-05 | 4.882 | 2.179 | 0.706 |
| 3 | FOLH1 | TC11001758.hg.1 | 1.525E-09 | 3.742E-12 | 10.790 | 17.737 | 0.580 |
| 4 | ANPEP | TC15001837.hg.1 | 2.267E-06 | 2.663E-08 | 7.319 | 9.032 | 0.471 |
| 5 | DPP4 | TC02002478.hg.1 | 8.960E-09 | 3.176E-11 | 9.894 | 15.649 | 0.463 |
| 6 | CPE | TC04000829.hg.1 | 9.100E-05 | 3.355E-06 | 5.619 | 4.272 | 0.448 |
| 7 | CPXM2 | TC10001721.hg.1 | 1.612E-04 | 6.945E-06 | 5.369 | 3.558 | 0.441 |
| 8 | CTSK | TC01003211.hg.1 | 2.421E-04 | 1.172E-05 | 5.188 | 3.045 | 0.417 |
| 9 | TLL1 | TC04000833.hg.1 | 6.146E-14 | 2.179E-17 | 16.837 | 29.238 | 0.409 |
| 10 | CMA1 | TC14000979.hg.1 | 2.781E-08 | 1.144E-10 | 9.378 | 14.394 | 0.392 |
| 11 | NAALAD2 | TC11002820.hg.1 | 1.753E-14 | 4.226E-18 | 10.667 | 14.598 | 0.385 |
| 12 | CES1 | TC16001128.hg.1 | 3.690E-05 | 1.022E-06 | 6.030 | 5.439 | 0.385 |
| 13 | C1S | TC12003224.hg.1 | 3.185E-05 | 8.374E-07 | 6.100 | 5.636 | 0.377 |
| 14 | TINAGL1 | TC01000411.hg.1 | 8.413E-10 | 1.825E-12 | 11.100 | 18.435 | 0.335 |
| 15 | DPYSL3 | TC05001909.hg.1 | 4.926E-04 | 2.985E-05 | 4.866 | 2.131 | 0.323 |
| 16 | ADAMTS1 | TC21000891.hg.1 | 4.844E-04 | 2.916E-05 | 3.397 | -1.465 | 0.316 |
| 17 | MASP1 | TC03003257.hg.1 | 1.445E-11 | 1.454E-14 | 13.262 | 22.950 | 0.311 |
| 18 | CAPN6 | TC0X001279.hg.1 | 1.004E-02 | 1.710E-03 | 2.548 | -3.636 | 0.297 |
| 19 | HTRA3 | TC04000087.hg.1 | 9.314E-05 | 3.455E-06 | 5.609 | 4.243 | 0.288 |
| 20 | ADAMTS5 | TC21000346.hg.1 | 5.675E-05 | 1.796E-06 | 5.482 | 3.881 | 0.283 |
| 21 | FAP | TC02002480.hg.1 | 3.088E-03 | 3.456E-04 | 4.008 | -0.245 | 0.281 |
| 22 | C1R | TC12001156.hg.1 | 2.151E-03 | 2.126E-04 | 4.181 | 0.224 | 0.271 |
| 23 | EPHX1 | TC01001856.hg.1 | 2.184E-04 | 1.032E-05 | 5.232 | 3.170 | 0.261 |
| 24 | ADAM3B | TC16001879.hg.1 | 1.927E-03 | 1.842E-04 | 4.231 | 0.362 | 0.234 |
| 25 | MMP3 | TC11002235.hg.1 | 7.775E-04 | 5.407E-05 | 3.655 | -1.037 | 0.230 |
| 26 | ADAMTS3 | TC04001277.hg.1 | 5.316E-05 | 1.643E-06 | 5.866 | 4.973 | 0.229 |
| 27 | C1RL | TC12001157.hg.1 | 3.627E-04 | 1.985E-05 | 5.007 | 2.530 | 0.225 |
| 28 | LIPE | TC19001573.hg.1 | 3.298E-07 | 2.371E-09 | 8.208 | 11.415 | 0.219 |
| 29 | USP46 | TC04001195.hg.1 | 1.315E-05 | 2.605E-07 | 6.508 | 6.785 | 0.213 |
| 30 | GFPT2 | TC05002146.hg.1 | 1.730E-02 | 3.571E-03 | 3.147 | -2.470 | 0.196 |

**Supplementary Table 3**

| **No** | **Probe Name** | **MD-MSC** | **MD-MSC** | **MD-MSC** | **WT-MSC** | **WT-MSC** | **WT-MSC** | **MD-MSC tumor-1** | **MD-MSC tumor-2** | **MD-MSC tumor-3** | **MD-MSC tumor-4** | **MD-MSC tumor-5** | **MD-MSC tumor-6** |
| --- | --- | --- | --- | --- | --- | --- | --- | --- | --- | --- | --- | --- | --- |
| 1 | ADAM15 | 2.71 | 2.72 | 2.63 | 2.84 | 2.86 | 2.67 | 2.66 | 2.78 | 2.74 | 2.53 | 2.79 | 2.75 |
| 2 | ADAM17 | 3.05 | 3.03 | 3.03 | 2.75 | 2.7 | 2.54 | 3 | 3.15 | 3.06 | 2.87 | 3.15 | 3.08 |
| 3 | ADAM28 | 0.69 | 0.66 | 0.84 | 1.1 | 0.87 | 0.82 | 1.15 | 1.15 | 1 | 0.88 | 0.89 | 1.33 |
| 4 | ADAM8 | 1.61 | 1.66 | 1.29 | 1.4 | 1.37 | 1.63 | 2.39 | 2.3 | 2.36 | 2.9 | 2.49 | 2.6 |
| 5 | ADAM9 | 3.41 | 3.39 | 3.35 | 3.6 | 3.62 | 3.5 | 3.36 | 3.44 | 3.42 | 3.22 | 3.44 | 3.44 |
| 6 | ADAMTS1 | 2.78 | 2.83 | 2.73 | 3.24 | 3.22 | 3.5 | 2.86 | 3.11 | 2.89 | 2.76 | 2.84 | 3 |
| 7 | ADAMTS12 | 1.86 | 1.92 | 1.75 | 2.19 | 2.08 | 2.08 | 2.68 | 2.81 | 2.75 | 2.13 | 2.86 | 2.72 |
| 8 | ADAMTS8 | 0.49 | 0.89 | 0.99 | 0.93 | 0.93 | 1.09 | 2.18 | 2.04 | 2.04 | 1.62 | 2.09 | 1.89 |
| 9 | AGRN | 2.66 | 2.67 | 2.41 | 2.66 | 2.64 | 2.74 | 2.65 | 2.8 | 2.71 | 2.26 | 2.69 | 2.64 |
| 10 | CASP8 | 2.24 | 2.23 | 2.23 | 2.16 | 2.07 | 2.5 | 2.7 | 2.75 | 2.77 | 2.59 | 2.71 | 2.71 |
| 11 | CCL1 | 0.69 | 1 | 0.59 | 0.85 | 1.09 | 1.02 | 1.07 | 1.43 | 1.1 | 1.04 | 1.02 | 0.85 |
| 12 | CCL2 | 3.22 | 3.21 | 3.29 | 2.17 | 2.28 | 2.81 | 3.29 | 3.25 | 3.34 | 3.5 | 3.15 | 3.32 |
| 13 | CCL4 | 0.09 | 0.49 | 0.95 | 0.45 | 0.87 | 0.98 | 1.45 | 1.9 | 1.95 | 2.23 | 2.03 | 1.79 |
| 14 | CCL7 | 2.69 | 2.7 | 2.78 | 2.05 | 2.08 | 2.2 | 2.57 | 2.76 | 2.68 | 2.89 | 2.46 | 2.73 |
| 15 | CD44 | 1.4 | 1.49 | 1.52 | 1.51 | 1.63 | 1.52 | 1.75 | 1.78 | 1.63 | 1.65 | 1.75 | 1.81 |
| 16 | CDH1 | 0.69 | 0 | 0.47 | 1.19 | 1.04 | 1.05 | 1.96 | 2.08 | 1.88 | 1.27 | 1.61 | 1.75 |
| 17 | CLDN3 | 0.93 | 1.14 | 0.69 | 1.23 | 1.43 | 1.18 | 1.45 | 1.62 | 1.42 | 1.2 | 1.62 | 1.5 |
| 18 | CMA1 | 1.33 | 1.44 | 1.11 | 1.26 | 1.39 | 1.18 | 2.39 | 2.58 | 2.98 | 1.81 | 2.52 | 2.7 |
| 19 | COL1A1 | 3.87 | 3.87 | 3.74 | 4.44 | 4.44 | 4.13 | 4.81 | 5.03 | 4.78 | 4.41 | 4.87 | 4.88 |
| 20 | COL1A2 | 3.55 | 3.56 | 3.46 | 3.86 | 3.86 | 3.5 | 3.95 | 4.19 | 3.93 | 3.57 | 4.04 | 4.02 |
| 21 | COL3A1 | 3.61 | 3.61 | 3.55 | 4.2 | 4.2 | 3.48 | 4.49 | 4.79 | 4.5 | 3.89 | 4.58 | 4.69 |
| 22 | COL4A1 | 2.56 | 2.58 | 2.4 | 3.09 | 3.14 | 3.03 | 3.08 | 3.24 | 3.13 | 2.53 | 3.14 | 3.11 |
| 23 | COL4A2 | 3.04 | 3.05 | 2.92 | 3.53 | 3.55 | 3.41 | 3.5 | 3.64 | 3.49 | 2.93 | 3.54 | 3.53 |
| 24 | COL4A6 | 0.79 | 0.66 | 0.77 | 1.38 | 1.39 | 1.67 | 1.18 | 1.43 | 0.8 | 0.92 | 1.35 | 1.26 |
| 25 | COL5A1 | 2.38 | 2.39 | 2.2 | 2.59 | 2.6 | 2.48 | 2.78 | 2.93 | 2.73 | 2.38 | 2.77 | 2.85 |
| 26 | COL5A2 | 4 | 4.01 | 3.97 | 3.76 | 3.75 | 3.57 | 4.31 | 4.42 | 4.31 | 3.84 | 4.34 | 4.34 |
| 27 | COL6A1 | 2.87 | 2.89 | 2.78 | 3.59 | 3.6 | 3.15 | 3.89 | 3.99 | 3.78 | 3.31 | 3.83 | 3.77 |
| 28 | COL6A2 | 2.7 | 2.71 | 2.67 | 3.51 | 3.52 | 3.09 | 3.87 | 3.98 | 3.77 | 3.23 | 3.86 | 3.81 |
| 29 | COL6A3 | 2.85 | 2.86 | 2.53 | 2.98 | 2.98 | 3.24 | 3.52 | 3.74 | 3.38 | 3.17 | 3.27 | 3.56 |
| 30 | COL7A1 | 1.46 | 1.33 | 1.14 | 1.45 | 1.45 | 2.35 | 2.14 | 2.32 | 1.77 | 1.74 | 2.23 | 1.83 |
| 31 | CXCL11 | 0.49 | 0.79 | 0.59 | 0.93 | 0.99 | 0.58 | 1.87 | 2.1 | 1.81 | 2.29 | 1.8 | 1.7 |
| 32 | FGF2 | 1.36 | 1.33 | 1.41 | 1.98 | 1.95 | 2.3 | 2 | 2.03 | 2.11 | 1.72 | 2.14 | 2.06 |
| 33 | FN1 | 4.4 | 4.41 | 4.04 | 3.94 | 3.94 | 3.66 | 4.23 | 4.32 | 4.04 | 4 | 4.1 | 4.15 |
| 34 | HAS1 | 0.27 | 0.73 | 0.59 | 0.93 | 0.87 | 1.3 | 1.24 | 1.41 | 1.4 | 1.82 | 1.32 | 1.46 |
| 35 | HGF | 2.35 | 2.37 | 2.24 | 2.2 | 2.21 | 2.02 | 2.75 | 2.83 | 2.68 | 2.67 | 2.75 | 2.79 |
| 36 | HIF1A | 3.58 | 3.58 | 3.53 | 3.46 | 3.46 | 3.37 | 3.29 | 3.4 | 3.33 | 3.22 | 3.39 | 3.38 |
| 37 | IBSP | 0.27 | 0.36 | 0.69 | 2.47 | 2.44 | 0.93 | 0.88 | 0.85 | 0.1 | 0.4 | 0.59 | 0.85 |
| 38 | IFNG | 1.07 | 0.59 | 1.07 | 1.23 | 1.17 | 0.93 | 0.63 | 1.28 | 1.05 | 0.92 | 1.29 | 1.05 |
| 39 | IL6 | 2.11 | 2.13 | 2.18 | 1.4 | 1.45 | 2.28 | 1.37 | 1.49 | 1.4 | 1.22 | 1.51 | 1.79 |
| 40 | ITGA1 | 2.7 | 2.69 | 2.61 | 2.55 | 2.55 | 2.21 | 2.97 | 3.36 | 3.2 | 2.46 | 3.19 | 3.23 |
| 41 | ITGA11 | 0.83 | 0.59 | 0.89 | 2.74 | 2.7 | 1.67 | 1.52 | 1.66 | 1.21 | 1.07 | 1.63 | 1.81 |
| 42 | ITGA2 | 2.4 | 2.37 | 2.27 | 1.55 | 1.58 | 2.44 | 2.7 | 2.78 | 2.76 | 2.58 | 2.64 | 2.74 |
| 43 | ITGA3 | 3.15 | 3.15 | 3.04 | 2.04 | 1.97 | 2.76 | 2.65 | 2.67 | 2.74 | 2.63 | 2.65 | 2.72 |
| 44 | ITGA5 | 2.46 | 2.44 | 2.35 | 2.64 | 2.62 | 3.14 | 2.59 | 2.61 | 2.52 | 2.64 | 2.54 | 2.64 |
| 45 | ITGA6 | 3.19 | 3.19 | 3.21 | 2.8 | 2.79 | 3.34 | 3.26 | 3.35 | 3.27 | 2.97 | 3.3 | 3.3 |
| 46 | ITGA7 | 1.72 | 1.66 | 1.55 | 2.36 | 2.3 | 2.58 | 1.52 | 1.74 | 1.51 | 1.35 | 1.76 | 1.53 |
| 47 | ITGA8 | 1.96 | 1.95 | 1.92 | 2.66 | 2.69 | 1.84 | 2.18 | 2.08 | 2.14 | 2.15 | 2.13 | 2.16 |
| 48 | ITGA9 | 0.96 | 0.79 | 0.59 | 1.4 | 1.23 | 1.45 | 1.96 | 2.2 | 2.12 | 1.72 | 2.21 | 2.19 |
| 49 | ITGAM | 1.37 | 1.19 | 1.35 | 1.26 | 1.04 | 1.21 | 3 | 3.18 | 2.94 | 2.84 | 3.08 | 3.08 |
| 50 | ITGB1 | 4.05 | 4.04 | 4.03 | 4.18 | 4.17 | 4.46 | 4.14 | 4.19 | 4.14 | 4 | 4.23 | 4.15 |
| 51 | ITGB1BP1 | 2.78 | 2.81 | 2.91 | 2.95 | 2.94 | 2.84 | 2.74 | 2.79 | 2.74 | 2.64 | 2.77 | 2.79 |
| 52 | ITGB2 | 1.29 | 1.36 | 1.07 | 0.75 | 0.69 | 0.45 | 2.31 | 2.51 | 2.33 | 2.31 | 2.58 | 2.46 |
| 53 | ITGB3 | 1.67 | 1.62 | 1.54 | 1.23 | 1.5 | 2.24 | 1.56 | 1.9 | 1.83 | 1.44 | 1.78 | 1.87 |
| 54 | ITGB4 | 2.2 | 2.15 | 2.02 | 1.92 | 1.95 | 2 | 2.44 | 2.49 | 2.47 | 2.48 | 2.44 | 2.57 |
| 55 | ITGB6 | 0.69 | 0.49 | 0.84 | 0.99 | 0.99 | 1.02 | 0.94 | 1.15 | 1.21 | 0.83 | 1.02 | 1.21 |
| 56 | ITGB7 | 1.34 | 1.41 | 1.52 | 1.19 | 0.99 | 1.73 | 1.63 | 1.91 | 1.83 | 2.18 | 1.55 | 1.88 |
| 57 | ITGB8 | 2.11 | 2.06 | 1.99 | 2.33 | 2.29 | 2.23 | 2.22 | 2.43 | 2.25 | 1.81 | 2.29 | 2.27 |
| 58 | KLK3 | 1.27 | 1.12 | 1.31 | 1.45 | 1.17 | 1.15 | 1.35 | 1.5 | 1.3 | 1.15 | 1.37 | 1.31 |
| 59 | LAMA1 | 1.37 | 1.44 | 0.89 | 1.4 | 1.43 | 1.02 | 1.18 | 1.39 | 1.24 | 0.88 | 1.42 | 1.15 |
| 60 | LAMA3 | 0.99 | 0.79 | 0.89 | 0.63 | 1.13 | 1.09 | 1.56 | 1.88 | 1.68 | 1.37 | 1.59 | 1.6 |
| 61 | LAMA4 | 3.08 | 3.09 | 2.98 | 2.95 | 2.96 | 2.78 | 3.25 | 3.41 | 3.31 | 2.81 | 3.29 | 3.33 |
| 62 | LAMA5 | 2.87 | 2.88 | 2.42 | 1.1 | 1.47 | 1.69 | 2.3 | 2.66 | 2.45 | 2.16 | 2.41 | 2.42 |
| 63 | LAMB3 | 0.57 | 0.49 | 0.47 | 1.26 | 1.04 | 0.82 | 1.49 | 1.3 | 1.3 | 1 | 1.46 | 1.29 |
| 64 | LAMC1 | 3.53 | 3.52 | 3.34 | 3.19 | 3.21 | 3.15 | 3.54 | 3.7 | 3.54 | 3.3 | 3.51 | 3.59 |
| 65 | LAMC2 | 0.83 | 0.93 | 0.69 | 1.15 | 1.45 | 1.12 | 1.65 | 1.56 | 1.5 | 1.2 | 1.61 | 1.47 |
| 66 | MAPK1 | 3.27 | 3.25 | 3.2 | 3.34 | 3.35 | 3.29 | 3.3 | 3.38 | 3.33 | 3.24 | 3.36 | 3.31 |
| 67 | MMP1 | 1.34 | 1.14 | 1.25 | 1.33 | 1.2 | 1.57 | 1.33 | 1.19 | 1.24 | 1.25 | 1.32 | 1.39 |
| 68 | MMP10 | 0.39 | 0.66 | 0.84 | 0.99 | 0.79 | 2.63 | 1.03 | 1.07 | 0.94 | 1.15 | 1.19 | 0.85 |
| 69 | MMP12 | 0.69 | 0.79 | 0 | 0.75 | 0.79 | 1.36 | 2.1 | 1.9 | 2.25 | 2.66 | 1.63 | 2.08 |
| 70 | MMP13 | 0.74 | 0.79 | 0.29 | 2.42 | 2.39 | 2.49 | 1.79 | 1.58 | 1.33 | 1.35 | 1.56 | 1.6 |
| 71 | MMP14 | 2.96 | 2.98 | 2.93 | 3.46 | 3.46 | 3.27 | 3.5 | 3.73 | 3.5 | 3.2 | 3.68 | 3.57 |
| 72 | MMP17 | 1.94 | 1.91 | 1.92 | 2.14 | 2.1 | 2.57 | 2.32 | 2.33 | 2.44 | 1.81 | 2.36 | 2.29 |
| 73 | MMP2 | 3.45 | 3.42 | 3.44 | 3.48 | 3.5 | 3.17 | 3.56 | 3.86 | 3.71 | 3.48 | 3.76 | 3.74 |
| 74 | MMP24 | 0.87 | 0.66 | 0.89 | 0.93 | 1.04 | 1.21 | 0.99 | 0.77 | 1.05 | 0.7 | 0.96 | 0.91 |
| 75 | MMP3 | 1.46 | 1.28 | 1.44 | 2.79 | 2.79 | 3.7 | 2.29 | 1.96 | 2.02 | 1.67 | 2.1 | 2.65 |
| 76 | MMP9 | 2.09 | 2.1 | 2.02 | 0.99 | 0.69 | 0.98 | 3.3 | 3.19 | 3.25 | 3.03 | 3.09 | 3.12 |
| 77 | NF2 | 2.93 | 2.91 | 2.85 | 2.84 | 2.79 | 2.82 | 2.82 | 2.97 | 2.94 | 2.66 | 2.92 | 2.91 |
| 78 | NRP1 | 2.69 | 2.73 | 2.67 | 3.45 | 3.45 | 2.99 | 3.31 | 3.48 | 3.4 | 2.96 | 3.47 | 3.42 |
| 79 | PDGFA | 3.21 | 3.21 | 3.25 | 2.79 | 2.79 | 3.45 | 3.41 | 3.45 | 3.48 | 3.23 | 3.54 | 3.48 |
| 80 | PDGFC | 2.87 | 2.86 | 2.78 | 2.75 | 2.76 | 2.62 | 2.53 | 2.7 | 2.69 | 2.08 | 2.89 | 2.73 |
| 81 | PDGFRB | 2.39 | 2.36 | 2.12 | 2.65 | 2.59 | 2.3 | 2.78 | 3.06 | 2.91 | 2.25 | 2.86 | 2.87 |
| 82 | PLAU | 0.93 | 0.73 | 1.03 | 2.08 | 2.06 | 3.01 | 2.79 | 2.8 | 2.8 | 2.75 | 2.87 | 2.9 |
| 83 | PRSS22 | 0.93 | 0.49 | 0.99 | 1.1 | 1.04 | 1.18 | 1.26 | 1.49 | 1.38 | 0.96 | 1.44 | 1.44 |
| 84 | PRSS8 | 0.79 | 0.89 | 0.84 | 1.33 | 1.2 | 1.18 | 1.55 | 1.75 | 1.48 | 0.83 | 1.58 | 1.47 |
| 85 | RELN | 0.69 | 0.73 | 0.84 | 1.05 | 0.93 | 1.21 | 0.81 | 1.69 | 1.27 | 0.52 | 1.32 | 1.21 |
| 86 | SERPINA1 | 0.49 | 1.03 | 0.89 | 1.26 | 0.87 | 0.98 | 1.35 | 1.53 | 1.48 | 1 | 1.48 | 1.33 |
| 87 | SERPINE1 | 2.14 | 2.1 | 2.04 | 2.7 | 2.7 | 3.49 | 2.85 | 2.74 | 2.7 | 2.71 | 2.84 | 2.91 |
| 88 | SERPINH1 | 4.03 | 4.02 | 4.06 | 4.16 | 4.17 | 4.13 | 4.13 | 4.28 | 4.23 | 4.05 | 4.3 | 4.26 |
| 89 | TGFB1 | 2.45 | 2.45 | 2.33 | 2.38 | 2.38 | 2.85 | 2.83 | 2.85 | 2.82 | 2.58 | 2.86 | 2.84 |
| 90 | TIMP1 | 2.98 | 3 | 3.03 | 3.52 | 3.52 | 4.27 | 3.34 | 3.26 | 3.27 | 3.41 | 3.39 | 3.32 |
| 91 | TIMP2 | 3.73 | 3.75 | 3.7 | 3.85 | 3.86 | 3.65 | 3.56 | 3.74 | 3.7 | 3.34 | 3.79 | 3.7 |
| 92 | TIMP4 | 0.63 | 1 | 0.77 | 2.06 | 2.11 | 1.09 | 1.18 | 1.3 | 1.18 | 1.07 | 1.16 | 1.46 |
| 93 | TMPRSS2 | 0.27 | 0.59 | 0.95 | 0.63 | 1.2 | 0.82 | 0.94 | 1.37 | 0.88 | 0.83 | 1.35 | 1.33 |
| 94 | TMPRSS4 | 0.09 | 0.59 | 0.59 | 0.99 | 0.93 | 0.67 | 1.45 | 1.33 | 1.05 | 0.96 | 1.5 | 1.26 |
| 95 | TMPRSS6 | 0.63 | 0.59 | 0.29 | 0.63 | 0.87 | 0.58 | 0.88 | 1.11 | 0.8 | 0.7 | 0.81 | 0.71 |
| 96 | TNF | 1.34 | 1.41 | 1.2 | 1.19 | 1.09 | 1.09 | 1.76 | 1.83 | 1.77 | 1.71 | 1.75 | 1.65 |
| 97 | TPSB2 | 0.83 | 0.89 | 0.47 | 0.99 | 0.99 | 1.02 | 2.13 | 2.27 | 2.62 | 1.45 | 2.13 | 2.38 |
| 98 | TPSD1 | 1.31 | 1.38 | 1.37 | 1.19 | 1.32 | 1.28 | 2.48 | 2.6 | 2.36 | 1.56 | 1.92 | 2.27 |
| 99 | VEGFA | 3.33 | 3.33 | 3.19 | 2.89 | 2.91 | 3.08 | 2.87 | 2.65 | 2.93 | 3.31 | 2.85 | 2.92 |
| 100 | VEGFB | 3.04 | 3.01 | 3.07 | 2.95 | 2.98 | 2.82 | 2.92 | 2.97 | 3.02 | 2.93 | 3.04 | 2.98 |
| 101 | Abcf1 | 2.61 | 2.62 | 2.52 | 2.45 | 2.45 | 2.43 | 2.48 | 2.55 | 2.5 | 2.43 | 2.45 | 2.45 |
| 102 | Gapdh | 3.63 | 3.64 | 3.65 | 3.67 | 3.67 | 3.75 | 3.78 | 3.66 | 3.79 | 3.9 | 3.78 | 3.81 |
| 103 | Hprt | 2.53 | 2.52 | 2.61 | 2.66 | 2.65 | 2.59 | 2.51 | 2.56 | 2.49 | 2.45 | 2.54 | 2.52 |
| 104 | Tubb | 2.6 | 2.61 | 2.64 | 2.69 | 2.67 | 3.15 | 3.42 | 3.44 | 3.39 | 3.34 | 3.36 | 3.37 |

**Supplementary Table 4**

|  | **DEGs in implanted tumor vs. WT-MSC** | **Fold change** | **Log2 fold change** | **Concordant (Y/N)** |
| --- | --- | --- | --- | --- |
| 1 | MMP-9 | 15.120 | 3.918 | Y |
| 2 | ITGAM | 5.502 | 2.460 | Y |
| 3 | SERPINH1 | 4.584 | 2.197 | N |
| 4 | ITGB4 | 2.701 | 1.433 | Y |
| 5 | ITGB2 | 2.176 | 1.122 | Y |
| 6 | NF2 | 1.830 | 0.872 | Y |
| 7 | TNF | 2.450 | 1.293 | Y |
| 8 | CCL7 | 1.745 | 0.803 | Y |
| 9 | ADAMTS8 | 1.411 | 0.497 | Y |
| 10 | MAPK | 0.901 | -0.150 | Y |
| 11 | ITGB8 | 0.845 | -0.243 | Y |
| 12 | ITGBP1BP | 0.795 | -0.330 | Y |
| 13 | ADAM8 | 0.626 | -0.677 | N |
| 14 | PDGFC | 0.551 | -0.859 | Y |
| 15 | ADAM9 | 0.194 | -2.363 | Y |
| 16 | CDH1 | 0.192 | -2.383 | N |
| 17 | ITGA9 | 0.096 | -3.380 | N |
| 18 | ADAMTS12 | 0.094 | -3.410 | N |
| 19 | ARGN | 0.072 | -3.787 | Y |
| 20 | ITGA7 | 0.057 | -4.130 | Y |
| 21 | ADAMTS1 | 0.012 | -6.387 | Y |
| 22 | MMP-13 | 0.010 | -6.644 | Y |
| 23 | NF2 | 0.011 | -6.506 | Y |
| 24 | HIF1A | 1.27 | 0.345 | N |

|  | **DEGs in MD-MSC vs. WT-MSC** | **Fold change** | **Log2 fold change** | **Concordant (Y/N)** |
| --- | --- | --- | --- | --- |
| 1 | MMP-9 | 15.12 | 3.92 | Y |
| 2 | ADAM17 | 6.6521 | 2.734 | Y |
| 3 | ITGAM | 5.502 | 2.460 | Y |
| 4 | VEGEB | 4.1358 | 2.048 | Y |
| 5 | COL5A2 | 3.9134 | 1.968 | Y |
| 6 | CCL2 | 3.1298 | 1.646 | Y |
| 7 | COL4A2 | 2.9714 | 1.571 | N |
| 8 | ITGB4 | 2.701 | 1.433 | Y |
| 9 | TNF | 2.45 | 1.293 | Y |
| 10 | ITGB2 | 2.176 | 1.122 | Y |
| 11 | FN1 | 2.1471 | 1.102 | Y |
| 12 | ITGA3 | 2.1284 | 1.090 | Y |
| 13 | CCL7 | 1.745 | 0.803 | Y |
| 14 | LAMC1 | 1.5712 | 0.652 | Y |
| 15 | HIF1A | 1.27 | 0.345 | Y |
| 16 | LAMC5 | 1.0126 | 0.018 | Y |
| 17 | PDGFC | 0.551 | -0.860 | N |
| 18 | MMP3 | 0.4447 | -1.169 | Y |
| 19 | COL4A1 | 0.2714 | -1.882 | Y |
| 20 | ITGA9 | 0.096 | -3.381 | Y |
| 21 | ITGA7 | 0.057 | -4.133 | N |
| 22 | HGF | 0.0458 | -4.449 | N |
| 23 | LAM4A | 0.0351 | -4.832 | Y |
| 24 | ADAMTS1 | 0.012 | -6.381 | Y |
| 25 | NF2 | 0.011 | -6.506 | N |
| 26 | MMP-13 | 0.01 | -6.644 | Y |
| 27 | FGF2 | 0.005 | -7.644 | N |
| 28 | VEGFA | 0.0026 | -8.587 | Y |
| 29 | PRSS8 | 0.0021 | -8.895 | Y |
| 30 | COL4A6 | 0.0011 | -9.828 | Y |
| 31 | PLAU | 0.0009 | -10.118 | Y |
|  |  |  |  |  |
|  | **Not validated** |  |  |  |
| 32 | TPSD1 | n/a | n/a | n/a |

**Supplementary table 5**

| **ID** | **Age** | **Gender** | **Diameter (cm)** | **Adherent Tumor** | **Secreted MMP9 (ng/mL)** |
| --- | --- | --- | --- | --- | --- |
| 203 | 40 | F | 3.0 | No | 1.4 |
| 207 | 41 | F | 2.4 | No | 5.5 |
| 223 | 41 | F | 2.7 | No | 0 |
| 227 | 56 | M | 2.4 | No | 6.02 |
| 202 | 74 | M | 2.4 | No | 0.3 |
| 235 | 51 | M | 3.1 | Yes | 17.1 |
| 210 | 57 | M | 2.5 | Yes | 29.2 |
| 226 | 63 | M | 2.3 | Yes | 37.8 |
| 240 | 51 | F | 3.0 | Yes | 63.5 |
| 243 | 60 | M | 2.3 | Yes | 289.5 |

**Supplementary Table 6**

| **Number** | **Age** | **Gender** | **Sample** | **Tumor Diameter (mm)** | **Adherent** | **Cystic** | **Extent of resection** |
| --- | --- | --- | --- | --- | --- | --- | --- |
| **VS210** | 57 | M | VS | 25 | 1 | 0 | 1 |
| **VS211** | 62 | F | VS | 37 | 1 | 1 | 1 |
| **VS215** | 71 | M | VS | 49 | 1 | 1 | 1 |
| **VS201** | 59 | F | VS | 11 | 0 | 0 | 0 |
| **VS218** | 46 | M | VS | 17 | 0 | 0 | 0 |
| **VS217** | 70 | F | VS | 14 | 0 | 0 | 0 |
| **HD-16** | 56 | M | HD | N/A | N/A | N/A | N/A |
| **HD-31** | 61 | F | HD | N/A | N/A | N/A | N/A |
| **HD-24** | 68 | M | HD | N/A | N/A | N/A | N/A |
| **HD-36** | 59 | F | HD | N/A | N/A | N/A | N/A |
| **HD-26** | 46 | M | HD | N/A | N/A | N/A | N/A |
| **HD-30** | 70 | F | HD | N/A | N/A | N/A | N/A |

**Supplementary Table 7**

**Protease-VS vs. HD**

| **Gene Symbol** | **Gene Name** | **UniProt** | **Gene ID** | **Protease Type** | **median FC** | **p-val** |
| --- | --- | --- | --- | --- | --- | --- |
| MMP9 | Matrix metalloproteinase-9 | P14780 | 4318 | M | 2.817 | 0.022 |
| MMP19 | Matrix metalloproteinase-19 | Q99542 | 4327 | M | 2.270 | 0.007 |
| CELA2A | Chymotrypsin-like elastase family member 2A | P08217 | 63036 | S | 2.250 | 0.049 |
| ADAM9 | Disintegrin and metalloproteinase domain-containing protein 9 | Q13443 | 8754 | M | 2.097 | 0.001 |
| ELANE | Neutrophil elastase | P08246 | 1991 | S | 1.771 | 0.007 |
| CTSH | Cathepsin H | P09668 | 1512 | C | 1.704 | 0.000 |
| IHH | Indian hedgehog protein | Q14623 | 3549 | N/A | 1.645 | 0.016 |
| CTSB | Cathepsin B | P07858 | 1508 | C | 1.644 | 0.008 |
| PSMA5 | Proteasome subunit alpha type-5 | P28066 | 5686 | N/A | 1.597 | 0.008 |
| HGF | Hepatocyte growth factor | P14210 | 3082 | N/A | 1.512 | 0.021 |
| DPEP1 | Dipeptidase 1 | P16444 | 1800 | M | 1.371 | 0.034 |
| PROC | Vitamin K-dependent protein C | P04070 | 5624 | S | 1.360 | 0.005 |
| BCHE | Cholinesterase | P06276 | 590 | N/A | 1.301 | 0.025 |
| PSMB1 | Proteasome subunit beta type-1 | P20618 | 5689 | M | 1.283 | 0.037 |
| KLK4 | Kallikrein-4 | Q9Y5K2 | 9622 | S | 1.274 | 0.000 |
| KLK5 | Kallikrein-5 | Q9Y337 | 25818 | S | 1.253 | 0.024 |
| F7 | Coagulation factor VII | P08709 | 2155 | S | 1.251 | 0.020 |
| DPP10 | Inactive dipeptidyl peptidase 10 | Q8N608 | 57628 | S | 1.230 | 0.002 |
| C1R | Complement C1r subcomponent | P00736 | 715 | S | 1.223 | 0.003 |
| USP11 | Ubiquitin carboxyl-terminal hydrolase 11 | P51784 | 8237 | C | 1.214 | 0.006 |

**Supplementary Table 7**

**Protease- Adherent vs. HD**

| **Gene Symbol** | **Gene Name** | **UniProt** | **Gene ID** | **median FC** | **p-val** |
| --- | --- | --- | --- | --- | --- |
| MMP9 | Matrix metalloproteinase-9 | P14780 | 4318 | 6.913 | 0.002 |
| ADAM17 | Disintegrin and metalloproteinase domain-containing protein 17:Cytoplasmic domain | P78536 | 6868 | 4.341 | 0.040 |
| MMP19 | Matrix metalloproteinase-19 | Q99542 | 4327 | 3.230 | 0.001 |
| ADAM9 | Disintegrin and metalloproteinase domain-containing protein 9 | Q13443 | 8754 | 2.193 | 0.000 |
| ELANE | Neutrophil elastase | P08246 | 1991 | 2.039 | 0.002 |
| PRTN3 | Myeloblastin | P24158 | 5657 | 2.023 | 0.019 |
| MMP8 | Neutrophil collagenase | P22894 | 4317 | 1.836 | 0.017 |
| CTSH | Cathepsin H | P09668 | 1512 | 1.808 | 0.005 |
| LTF | Lactotransferrin | P02788 | 4057 | 1.797 | 0.007 |
| IHH | Indian hedgehog protein | Q14623 | 3549 | 1.768 | 0.005 |
| PROC | Activated Protein C | P04070 | 5624 | 1.752 | 0.012 |
| REN | Renin | P00797 | 5972 | 1.732 | 0.005 |
| CTSO | Cathepsin O | P43234 | 1519 | 1.704 | 0.002 |
| CTSB | Cathepsin B | P07858 | 1508 | 1.684 | 0.026 |
| FOLH1 | Glutamate carboxypeptidase 2 | Q04609 | 2346 | 1.676 | 0.021 |
| HGF | Hepatocyte growth factor | P14210 | 3082 | 1.614 | 0.005 |
| PROC | Vitamin K-dependent protein C | P04070 | 5624 | 1.540 | 0.006 |
| TINAGL1 | Tubulointerstitial nephritis antigen-like | Q9GZM7 | 64129 | 1.498 | 0.018 |
| PSMB1 | Proteasome subunit beta type-1 | P20618 | 5689 | 1.472 | 0.014 |
| CFD | Complement factor D | P00746 | 1675 | 1.459 | 0.043 |

**Supplementary table 8**

| **Cohort** | **Variable** | **AUC** | **p-val** | **Sensitivity** | **Specificity** | **PPV** | **NPV** |
| --- | --- | --- | --- | --- | --- | --- | --- |
| All | 1.PTA | 0.54 | 0.6 |  |  |  |  |
| All | 2.Age | 0.55 | 0.32 |  |  |  |  |
| All | 3.WRS | 0.56 | 0.5 |  |  |  |  |
| All | 4.Growth | 0.63 | 0.095 |  |  |  |  |
| All | 5.BS+CSF+Cyst | 0.75 | 0.015 | 25.0 | 96.55 | 75 | 75.7 |
| All | 6.MMP9 | 0.78 | 0.004 | 37.5 | 89.3 | 66.7 | 71.4 |
| All | 7.TVol | 0.84 | <0.0001 | 56.3 | 93.1 | 81.8 | 79.4 |
| All | 5+6. MMP9+BS+CSF+Cyst | 0.84 | 0.0013 | 66.7 | 92.9 | 80 | 86.7 |
| All | 5+6+7. MMP9+TVol+BS+CSF+Cyst | 0.88 | 0.0001 | 66.7 | 96.4 | 88.9 | 87.1 |
| All | 6+7. MMP9+TVol | 0.91 | <0.0001 | 75 | 96.4 | 92.3 | 87.1 |
|  |  |  |  |  |  |  |  |
| **Cohort** | **Variable** | **AUC** | **p-val** | **Se** | **Sp** | **PPV** | **NPV** |
| >15 mm | 4.Growth | 0.48 | 0.2 |  |  |  |  |
| >15 mm | 3.WRS | 0.53 | 0.6 |  |  |  |  |
| >15 mm | 1.PTA | 0.54 | 0.8 |  |  |  |  |
| >15 mm | 2.Age | 0.56 | 0.91 |  |  |  |  |
| >15 mm | 5.BS+CSF+Cyst | 0.68 | 0.182 |  |  |  |  |
| >15 mm | 7.TVol | 0.82 | 0.0007 | 64.3 | 83.3 | 81.8 | 66.7 |
| >15 mm | 6.MMP9 | 0.85 | 0.0029 | 71.4 | 91.7 | 90.9 | 73.3 |
| >15 mm | 5+6. MMP9+BS+CSF+Cyst | 0.89 | 0.0055 | 80.0 | 83.3 | 80 | 83.3 |
| >15 mm | 6+7. MMP9+TVol | 0.93 | <0.0001 | 92.9 | 91.7 | 92.9 | 91.7 |
| >15 mm | 5+6+7. MMP9+TVol+BS+CSF+Cyst | 0.95 | 0.0004 | 80.0 | 100 | 100 | 85.7 |

**Supplementary Table 9**

**Human**

| Gene Name | Primer | Sequence (5’- 3’) |
| --- | --- | --- |
| MMP-9 | Forward | CACTGTCCACCCCTCAGAGC |
|  | Reverse | GCCACTTGTCGGCGATAAGG |
| NOTCH-1 | Forward | GGTGAACTGCTCTGAGGAGATC |
|  | Reverse | GGATTGCAGTCGTCCACGTTGA |
| NOTCH-2 | Forward | GTGCCTATGTCCATCTGGATGG |
|  | Reverse | AGACACCTGAGTGCTGGCACAA |
| IL-6 | Forward | CCAGTACCCCCAGGAGAAGAT |
|  | Reverse | GAGGATGTACCGAATTTGTTTGT |
| TNF-α | Forward | TCAGCAAGGACAGCAGAGG |
|  | Reverse | AGTATGTGAGAGGAAGAGAACC |
| IL-1β | Forward | CCTATTACAGTGGCAATGAGGAT |
|  | Reverse | AGTGGTGGTCGGAGATTCG |
| B2M | Forward | ACTGAATTCACCCCCACTGA |
|  | Reverse | CCTCCATGATGCTGCTTACA |

**Mouse**

| Gene Name | Primer | Sequence (5’- 3’) |
| --- | --- | --- |
| MMP-9 | Forward | GCTGACTACGATAAGGACGGCA |
|  | Reverse | TAGTGGTGCAGGCAGAGTAGGA |
| TIMP-1 | Forward | CGCAGCGAGGAGGTTTCTCAT |
|  | Reverse | GGCAGTGATGTGCAAATTTCC |
| TIMP-2 | Forward | CTCGCTGGACGTTGGAGGAAAGAA |
|  | Reverse | AGCCCATCTGGTACCTGTGGTTCA |
| TIMP-3 | Forward | CTCTGCAACTCCGACATCGTGAT |
|  | Reverse | CAGCAGGTACTGGTACTTGTTGAC |
| TIMP-4 | Forward | CACTACCATCTGAACTGTGGCTG |
|  | Reverse | GCTTTCGTTCCAACAGCCAGTC |
| CCL7 | Forward | GCAGTCTGAAGGCACAGCAA |
|  | Reverse | GGTTGGCACAGACCTGGAAC |
| ADAM8 | Forward | AGGATATTCAGCAGGTGTAGCAA |
|  | Reverse | TGCTAAAGGTATAGCAGGAGTCG |
| ADAMTS8 | Forward | AGAGGACAGGAAGCAGGACAA |
|  | Reverse | GGACACAAACCTCTTGCTTCTAGTT |
| ITGA9 | Forward | ACAGCACCTCAGTGTCACGATG |
|  | Reverse | TTCTGTGCCACATCAGCCGTCA |
| ADAMTS12 | Forward | GCCTTGACAATGACGTGGAGAAG |
|  | Reverse | CCTTCTTGACACAATGAGCAGCC |
| CDH1 | Forward | GGTCATCAGTGTGCTCACCTCT |
|  | Reverse | GCTGTTGTGCTCAAGCCTTCAC |
| ITGAM | Forward | TCCGGTAGCATCAACAACAT |
|  | Reverse | GGTGAAGTGAATCCGGAACT |
| TNF | Forward | ATGAGAAGTTCCCAAATGGC |
|  | Reverse | CTCCACTTGGTGGTTTGCTA |
| MAPK1 | Forward | CCTTCAGAGCACTCCAGAAAGT |
|  | Reverse | ACAACACCAAAAAGGCATCC |
| ADAM9 | Forward | TGACCATCCCAACGTACAGA |
|  | Reverse | TTCCAAAACTGGCATTCTCC |
| ITGB1BP1 | Forward | AAGTCTCGACCACGGATCAGCA |
|  | Reverse | GCATCTGTGGTCTTGAGTGCCA |
| ITGB4 | Forward | ACACAAGCTCCAGCAGACGAAG |
|  | Reverse | TCCACCTGCTTCTCTGTCAGCT |
| HIF1A | Forward | CCTGCACTGAATCAAGAGGTTGC |
|  | Reverse | CCATCAGAAGGACTTGCTGGCT |
| AGRN | Forward | AATGGCACGGACAATTTGC |
|  | Reverse | TATGAGGGTTTGTGGGGTGT |
| ADAMTS1 | Forward | AGTTACCTCCAATGCAGCTCT |
|  | Reverse | ATCCCGAGAGTGTCACACGTG |
| ITGA7 | Forward | GCCACTCTGCCTGTCCAATG |
|  | Reverse | GGAGGTGCTAAGGATGAGGTAGA |
| ITGB8 | Forward | ACTTCTCCTGTCCCTATCTCC |
|  | Reverse | ATCTGCCACCTTCACACTCC |
| SERPINH1 | Forward | ATGTTCTTTAAGCCACACTG |
|  | Reverse | TCGTCATAGTAGTTGTACAGG |
| PDGFC | Forward | AATGCTGAGCGACCACTCCATC |
|  | Reverse | TCGGGTCATGTTCAAGTCCAGC |
| ITGB2 | Forward | CTTTCCGAGAGCAACATCCAGC |
|  | Reverse | GTTGCTGGAGTCGTCAGACAGT |
| LAMC1 | Forward | CTGTAATGGGCACAGTGAGACC |
|  | Reverse | ACAAGGCTGGCAGTCAGAGGAG |
| FN1 | Forward | CGAAGCCGGGAAGAGCAAG |
|  | Reverse | CGTTCCCACTGCTGATTTATCTG |
| HGF | Forward | CACCCCTTGGGAGTATTGTG |
|  | Reverse | GGGACATCAGTCTCATTCACAG |
| LAMA4 | Forward | AAGAAACCTTAGGAGTTGGTTATGGA |
|  | Reverse | ATAAAACTTTGCCCGTTGAAATATG |
| VEGFB | Forward | ACTGGGCAACACCAAGTCCGAA |
|  | Reverse | CACATTGGCTGTGTTCTTCCAGG |
| COL5A2 | Forward | CTTGGAAGTCAAGTAGGA |
|  | Reverse | GTCCTTGTAAACCCTGAG |
| ADAM17 | Forward | TTTTCACGTTTGCAGTCTCC |
|  | Reverse | TCCACGGCCCATGTATTTAT |
| ITGA3 | Forward | ACGAGACCATCCTGTGTGAGCT |
|  | Reverse | GCAAGTTGTCCTGGTGACTCGA |
| CCL2 | Forward | GGGCCTGCTGTTCACAGTT |
|  | Reverse | CCAGCCTACTCATTGGGAT |
| LAMA5 | Forward | ACCCAAGGACCCACCTGTAG |
|  | Reverse | TCATGTGTGCGTAGCCTCTC |
| PRSS8 | Forward | CCCATCTGCCTCCCTGC |
|  | Reverse | CCATCCCGTGACAGTACAGTGA |
| COL4A2 | Forward | CGGGTGTGAAAAGACCTATCGG |
|  | Reverse | CTGGCATTCCTCTGACGCCTTT |
| MMP3 | Forward | CAGGAAGATAGCTGAGGACT |
|  | Reverse | TTCCAACTGCGAAGATCCAC |
| PLAU | Forward | TTGGGGGAGAATTCACTGAG |
|  | Reverse | GGCAGGCAGATGGTCTGTAT |
| FGF2 | Forward | GTACCTTGCTATGAAGGAAGATG |
|  | Reverse | ATCCGAGTTTATACTGCCCA |
| COL4A6 | Forward | AGGGTCTACTGACCCATTTGGC |
|  | Reverse | ATCCAGGCTGACCTATGTCTCC |
| NF2 | Forward | GCTCAGGACCTGGAGATGTATG |
|  | Reverse | CAGCCTGTTCTCAGGGTCATAG |
| MMP13 | Forward | GATGACCTGTCTGAGGAAGACC |
|  | Reverse | GCATTTCTCGGAGCCTGTCAAC |
| COL4A1 | Forward | ATGGCTTGCCTGGAGAGATAGG |
|  | Reverse | TGGTTGCCCTTTGAGTCCTGGA |
| VEGFA | Forward | CTGCTGTAACGATGAAGCCCTG |
|  | Reverse | CTGCTGTAACGATGAAGCCCTG |
| GAPDH | Forward | AACTTTGGCATTGTGGAAGG |
|  | Reverse | GGATGCAGGGATGATGTTCT |
| B2M | Forward | GTGACCCTGGTCTTTCTGGT |
|  | Reverse | GTATGTTCGGCTTCCCATTC |
